# Photoswitchable TRPC6 channel activators evoke distinct channel kinetics reflecting different gating behaviors

**DOI:** 10.1016/j.isci.2024.111008

**Published:** 2024-09-20

**Authors:** Maximilian Keck, Christian Hermann, Kyra Lützel, Thomas Gudermann, David B. Konrad, Michael Mederos y Schnitzler, Ursula Storch

**Affiliations:** 1 Walther Straub Institute of Pharmacology and Toxicology, Ludwig Maximilian University of Munich, 80336 Munich, Germany; 2 Department of Pharmacy, Ludwig Maximilian University of Munich, 81377 Munich, Germany; 3 Institute of Pharmacy, Clinical Pharmacy, University of Regensburg, 93053 Regensburg, Germany

**Keywords:** Biophysical chemistry, Biological sciences, Biophysics

## Abstract

The non-selective *transient receptor potential canonical* 6 (TRPC6) cation channels have several physiological and pathophysiological effects. They are activated by the lipid second messenger diacylglycerol (DAG) and by non-lipidic compounds such as GSK 1702934A (GSK). Advances in photopharmacology led to the development of photoswitchable activators such as PhoDAG, OptoDArG, and OptoBI-1 that can be switched ON and OFF with the spatiotemporal precision of light. We aimed to elucidate whether these photopharmaceuticals allow for a reliable determination of the ion channel current kinetics. We performed electrophysiological whole-cell measurements in the overexpression system and analyzed TRPC6 currents induced by photoswitching. We observed distinct activation, deactivation and inactivation current kinetics suggesting that each photoswitchable activator elicits a distinct active channel state. Notably, the current kinetics strongly depended on the intensity of the light source. Altogether, photopharmaceuticals are advantageous for an extended biophysical characterization of whole-cell currents and provide insight into their gating mechanism.

## Highlights

• Photoswitchable activators enable precise determination of current kinetics
• Maximally effective concentrations are prerequisites
• High-intensity and fast-switching light sources are essential
• Each photoswitchable activator evokes a distinct and specific gating behavior

## Graphical abstract

**Figure:**
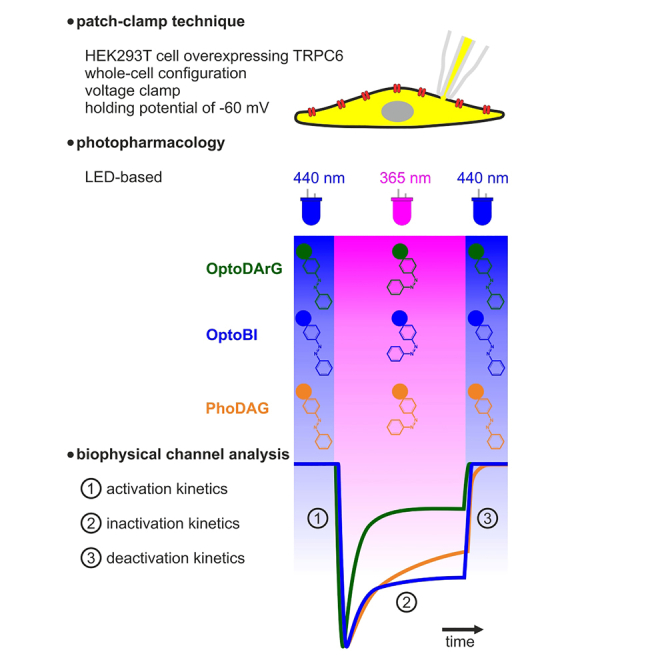

## Introduction

Transient receptor potential classical or canonical (TRPC) channels are non-selective cation channels that are permeable for sodium, potassium, and calcium ions and belong to the large family of TRP channels. TRPC channels are commonly regarded as receptor-operated cation channels that are activated following G_q/11_-protein coupled receptor activation. Receptor activation leads to activation of the phospholipase C which cleaves phosphoinositol-4,5-bisphosphate into the 2 s messengers inositol-1,4,5-trisphosphate (IP_3_) and diacylglycerol (DAG). DAG can then directly activate TRPC channels,^1^^,^^2^^,^^3^^,^^4^ leading to channel opening and cation influx, thus resulting in cellular effects. Accordingly, DAG serves as the endogenous activator of TRPC channels. However, the DAG sensitivity of TRPC4 and TRPC5 channels is regulated by C-terminal interaction with the adaptor proteins NHERF1 and NHERF2^3^ which involves C-terminal phosphorylation via protein kinase C.^3^^,^^5^^,^^6^

During the last decades several physiological as well as pathophysiological roles of TRPC channels have been identified (summarized in the study by Chen et al.^7^) e.g., TRPC channels play a role for the cardiovascular system, metabolism, immune system, kidney function, neurological function, and cancer. Furthermore, patient mutations in the gene coding for TRPC6 are associated with a chronic kidney disease, the focal segmental glomerulosclerosis (FSGS)^8^^,^^9^^,^^10^^,^^11^ which leads to podocyte damage and renal failure. Thus, TRPC channels are important for human health and disease and serve as potential drug targets.

To study TRPC channels *in vitro*, DAG derivatives are widely used as channel activators. Hereby, the membrane permeable DAG analog 1-oleoyl-2-acetyl-*sn*-glycerol (OAG) is often employed. Furthermore, non-lipidic TRPC channel activators are available such as the TRPC3/6 channel activator GSK 1702934A. Recent advances in photopharmacology have yielded new photoswitchable TRPC channel modulators that allow for the regulation of the channel function with the spatiotemporal precision of light.^12^^,^^13^^,^^14^^,^^15^^,^^16^ The basis for photoswitching of these compounds is the insertion of a photoswitchable azobenzene^12^^,^^13^^,^^16^ or an azoheteroarene^17^ moiety that allows for light-induced *cis* and *trans* isomerization. Hereby, UV light stabilizes the active *cis* configuration, while blue light produces the inactive *trans* configuration, thus enabling the regulation of channel activity simply by switching the light. Other photoswitchable TRP channel ligands include azo-vanilloids such as azCA4 and red-azCA4^18^^,^^19^ which target TRPV1 and “TRPswitch”, which targets TRPA1.^20^ Using these compounds, wash-in and wash-out effects that usually occur when applying channels activators via bath solution are circumvented, such that the determination of the channel kinetics becomes possible. At the moment three photoswitchable TRPC6 channel activators are available: PhoDAG, OptoDArG, and OptoBI-1.^12^^,^^13^^,^^14^^,^^15^^,^^16^

In this study we aimed at analyzing whether the use of different photopharmaceuticals allows for a valid and reproducible determination of the reliable kinetics of TRPC6 currents which might improve the biophysical analysis of whole-cell currents thus allowing to gain deeper insights into the gating of ion channels. Since TRPC6 channels emerged as potential drug targets, the elucidation of their activation and regulation mechanism is important to improve the design of novel, potent channel modulators that might be helpful for the treatment of TRPC6 channel-related diseases.

## Results

### OptoDArG-evoked TRPC6 current kinetics strongly depend on the intensity of the light source

TRPC6 channels are not only sensitive to the endogenous agonist DAG, but also to small molecule activators such as the non-selective and non-lipidic TRPC3 and TRPC6 channel activator GSK 1702934A (GSK). Performing a biophysical analysis of the current-voltage relations elicited by 1-oleoyl-2-acetyl-*sn*-glycerol (OAG; 100 μM), a membrane-permeable analog of DAG, or by GSK (10 μM) in their maximally effective concentrations and calculating the normalized slope conductance (NSC)^21^ to quantify differences between the individual curve progressions, we observed significant differences in the NSC curve progression mainly at negative potentials suggesting that OAG and GSK have different activation mechanisms and cause distinct active channel states (Figure S1;^21^). Next, we employed photopharmacology utilizing the photoswitchable GSK derivative OptoBI-1,^16^ which contains one photoswitchable azobenzene group, and the photoswitchable DAG derivative OptoDArG,^12^ a DAG analog, that contains two photoswitchable azobenzene groups. The chemical structures of the TRPC6 channel activators are displayed in Figure S2.

The photopharmaceuticals were also applied in their maximally effective concentrations. The inactive *trans* configuration was established by applying blue light and the active *cis* configuration by applying UV light. Photoswitching was performed with different light sources, with a xenon lamp emitting UV light of in the range of 355–375 nm with a mean wavelength of 365 nm and blue light in the range of 435–455 nm with a mean wavelength of 445 nm or with two light-emitting diodes (LEDs) emitting UV light in the range of 346–371 nm with a peak wavelength of 367 nm and blue light in the range of 418–447 nm with a peak wavelength of 442 nm.

**Figure 1:**
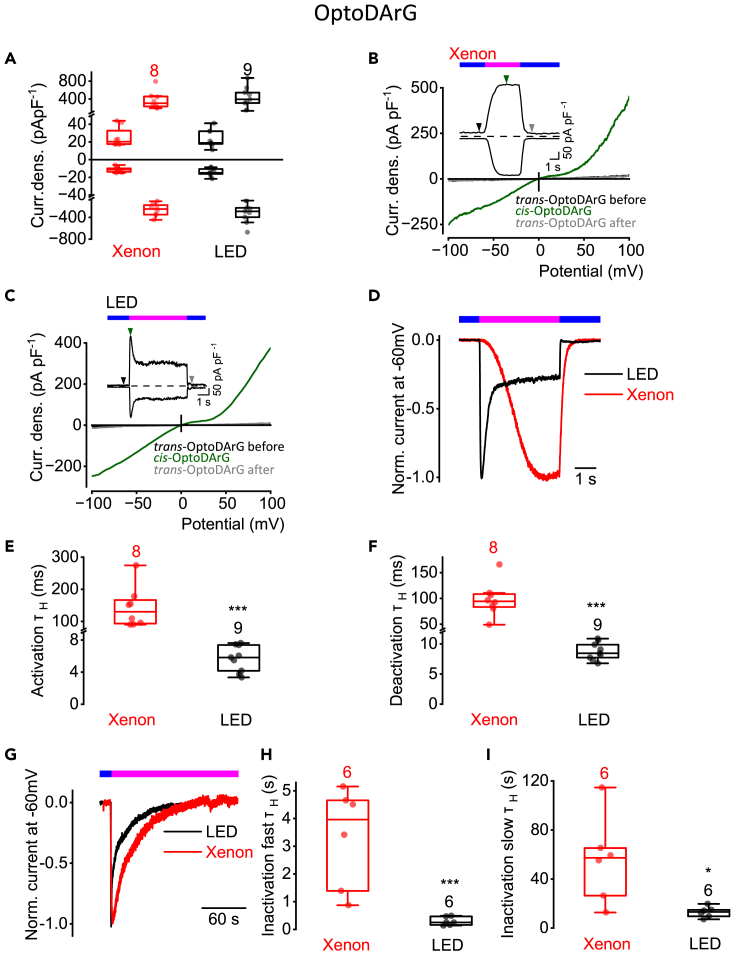
OptoDArG-evoked TRPC6 current kinetics strongly depend on the intensity of the light source Electrophysiological whole-cell measurements of TRPC6 overexpressing HEK293T cells in the presence of 30 μM OptoDArG. (A) Summaries of current densities (“Curr. dens.”) at potentials of ±100 mV evoked by light using a xenon lamp (red) or a LED (black). First small boxplots represent current densities in the presence of blue light (445 nm) which establishes *trans*-OptoDArG and second boxplots represent maximal current densities in the presence of UV light (365 nm) which establishes *cis*-OptoDArG. No significant differences between *trans* or *cis*-OptoDArG-induced current densities were observed using xenon or LED light source, respectively (Mann-Whitney U test). (B and C) Representative current density (“Curr. dens.”)-time courses ±100 mV and current density-voltage relations using xenon lamp (B) or LED (C) for photoswitching. The arrows indicate the time point at which the *trans-* and the maximal *cis*-OptoDArG-induced current density-voltage relations were selected. Bars over current density-time courses indicate the application of light of the wavelength 445 nm (blue) and of 365 nm (magenta). (D) Representative normalized time courses of TRPC6 inward currents at constant holding potential of −60 mV during photoswitching from blue light (blue bar) to UV light (magenta bar) and back to blue light (blue bar) using a xenon lamp (red) or LED (black). (E and F) Summaries of half-life time constants (τ_H_) of the activation (E) and deactivation (F) kinetics (∗∗∗*p* < 0.001; Mann-Whitney U test). (G) Representative normalized time courses of TRPC6 inward currents at constant holding potential of −60 mV induced by photoswitching from blue light to UV light using xenon lamp (red) or LED (black). The duration of light application (blue and magenta bar) is indicated. (H and I) Summaries of half-life time constants (τ_H_) of the fast (H) and slow (I) inactivation kinetics. (∗*p* < 0.05, ∗∗∗*p* < 0.001; Mann-Whitney U test). (E, F, H, and I) Data are displayed as boxplots and interquartile ranges. Numbers over boxplots indicate number of measured cells.

First, we performed electrophysiological whole-cell measurements with TRPC6 over-expressing HEK293T cells in the presence of 30 μM OptoDArG. To obtain current density-voltage relations and current densities at ±100 mV, consecutive voltage upramps from −100 to +100 mV were applied with a frequency of 50 Hz. Application of UV light for 4 s with the xenon lamp or with the LED resulted in comparable maximal TRPC6 current density increases at ±100 mV that were not significantly different (Figure 1A). OptoDArG caused almost symmetrical increases of the inward and outward CD. However, the current density-time courses strongly differed (Figures 1B and 1C). When applying UV light, the activation kinetics was much slower using a xenon lamp compared to the LED. However, the outward and inward currents at ±100 mV reached a plateau within 1–3 s of UV light application (Figure 1B). In contrast, using the 365 nm LED, the TRPC6 currents rapidly increased to maximal values in less than a second followed by an inactivation phase (Figure 1C). In both cases, switching to blue light resulted in fast current deactivations leading to rapid TRPC6 current decreases that reached the basal currents before application of UV light. These findings show that the TRPC6 channel was effectively switched OFF with blue light. However, the establishment of the *cis* configuration with UV light was less effective using the xenon lamp.

To gain further insights into the current kinetics, we proceeded to analyze TRPC6 inward currents, which were measured at a constant holding potential of −60 mV. Comparison of the current-time courses of the normalized currents elicited by photoswitching with the xenon lamp versus the LED in the presence of OptoDArG reveals marked differences in the activation kinetics (Figure 1D). The half-life time constants (τ_H_) of the activation, deactivation and inactivation kinetics were significantly faster when using the LED. τ_H_ were calculated using Equations 1, 2, 3, 4, and 5 and the parameters displayed in Table 1. The activation kinetics was 22-fold lower using LED light (5.8 ± 1.6 ms with LED and 130.0 ± 62.8 ms with xenon lamp) (Figure 1E). In addition, the deactivation kinetics was 11 times faster using LED light (8.4 ± 1.5 ms with LEDs and 94.4 ± 33.1 ms with xenon lamp (Figure 1F). To determine the inactivation kinetics, UV light was constantly applied until the TRPC6 current reached basal values (Figure 1G). The inactivation kinetics was biphasic showing a fast phase followed by a slow phase (Figures 1H and 1I). τ_H_ of the fast inactivation was 16-fold faster when using LED light (0.3 ± 0.2 s with LED and 4.0 ± 1.8 s with xenon lamp). The slow inactivation was 4-fold faster using LED light compared to the xenon lamp (13.1 ± 4.4 s with LEDs and 57.3 ± 35.5 s with xenon lamp). Thus, the xenon lamp was not sufficient for effective photoswitching of OptoDArG into its active *cis* and inactive *trans* configuration. To find out whether inactivation kinetics might depend on the current amplitudes, we correlated the fast and slow inactivation kinetics of OptoDArG and OptoBI-1 induced current densities that were elicited using LED or xenon lamp. However, we did not find any correlation (R^2^ was between 0.02 and 0.65) (Figure S3). Our findings strongly indicate that the intensity of the light source is crucial for the current kinetics. Since the maximal current densities at ±100 mV elicited by applying UV light using the two light sources xenon lamp and LED were not significantly different (Figure 1A), the observed differences in the current kinetics are independent of the current density amplitudes.

**Table 1:** Parameters and values for fit routine

Parameter	Value
MaxFunEvals	5000
MaxIter	10000
TolX	1∗10^−10^
TolFun	1∗10^−6^
Initial values activation	*a* = 0.01; τ_H_ = 1; *c* = 0.01
Initial values deactivation	*a* = 0.7; τ_H_ = 7; *c* = 0.1
Initial values inactivation	*a*_*1*_ = 1; τ_H1_ = 0.02; *c* = 0.1, *a*_*2*_ = 1, τ_H2_ = 3.8

Furthermore, we investigated the influence of lower LED light intensities on channel kinetics. To determine activation and deactivation kinetics, measurements were conducted by consecutively applying blue and UV light with increasing LED light intensities of 25%, 50%, and 100% to TRPC6 overexpressing HEK293T cells in the presence of OptoDArG (Figures S4A–S4D). Overall, activation and deactivation kinetics were significantly different. Using Dunn’s multiple comparison post hoc analysis, we particularly observed differences between the current kinetics observed with LED light intensities of 25% and 100%. To determine inactivation kinetics, LED light with 25% or 50% light intensity was applied to cells until currents reached values that occurred before application of UV light. Increasing light intensities resulted in faster inactivation kinetics, and the differences were most pronounced between 25% and 100% light intensity.

### OptoBI-1-induced TRPC6 current kinetics strongly rely on the intensity of the light source

**Figure 2:**
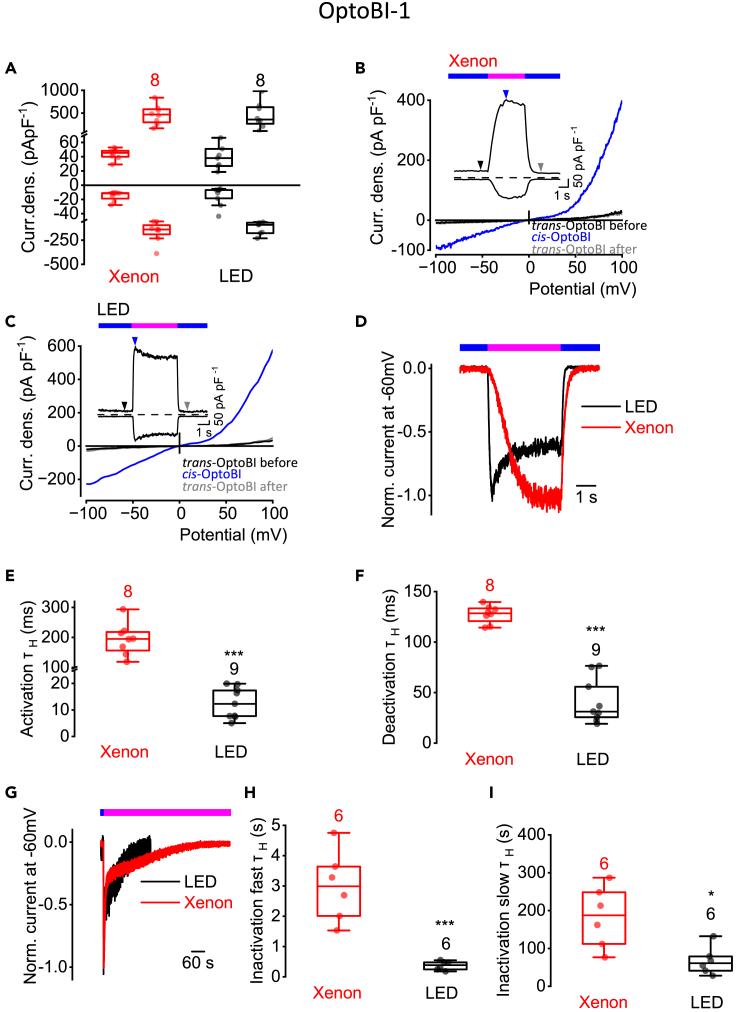
OptoBI-1-induced TRPC6 current kinetics strongly rely on the intensity of the light source Electrophysiological whole-cell measurements of TRPC6 overexpressing HEK293T cells in the presence of 10 μM OptoBI-1. (A) Summaries of current densities (“Curr. dens.”) at potentials of ±100 mV evoked by application of UV light using a xenon lamp (red) or a LED (black). First small boxplots represent current densities induced by *trans-* and second boxplots maximal current densities induced by *cis*-OptoBI-1. No significant differences between *trans*- or *cis*-OptoBI-1-induced current densities using xenon lamp or LED (Mann-Whitney U test). (B and C) Representative current density (“Curr. dens.”)-time courses ±100 mV and current density-voltage relations using xenon lamp (B) or LED (C) for photoswitching. The arrows indicate the time point at which the *trans*- and the maximal *cis*-OptoBI-1-induced current density-voltage relations were selected. Bars over current density-time courses indicate the application of light of the wavelength 445 nm (blue) and of 365 nm (magenta). (D) Representative normalized time courses of TRPC6 currents at constant holding potential of −60 mV during photoswitching from blue light (blue bar) to UV light (magenta bar) and back to blue light (blue bar) using a xenon lamp (red) or LED (black). (E and F) Summaries of half-life time constants (τ_H_) of the activation (E) and deactivation (F) kinetics (∗∗∗*p* < 0.001; Mann-Whitney U test). (G) Representative normalized time courses of TRPC6 currents at constant holding potential of −60 mV induced by photoswitching from blue light to UV light using xenon lamp (red) or LED (black). The duration of light application (blue and magenta bar) is indicated. (H and I) Summaries of half-life time constants (τ_H_) of the fast (H) and slow (I) inactivation kinetics (∗*p* < 0.05, ∗∗∗*p* < 0.001; Mann-Whitney U test). (E, F, H, and I) Data are displayed as boxplots and interquartile ranges. Numbers over boxplots indicate number of measured cells.

Next, we used the non-lipidic TRPC6 activator OptoBI-1 (10 μM) and again we performed photoswitching either with the xenon lamp or with LEDs. Exposure to UV light with both light sources caused maximal TRPC6 current density increases at ± 100 mV (Figure 2A) that were not significantly different. Interestingly, the OptoBI-1-induced CD at ±100 mV showed a pronounced outward rectification compared to the OptoDArG-induced CD. Using the xenon lamp, the current density maximum was reached within 2 s of UV light application (Figure 2B) but using the LED, the maximal current density was reached earlier within the first second of UV light application (Figure 2C). Furthermore, in both cases, blue light caused fast TRPC6 current deactivations. Interestingly, in contrast to *cis*-OptoDArG, *cis*-OptoBI-1 elicited TRPC6 current increases that reached a maximum followed by an inactivation phase. To determine the current kinetics, the normalized TRPC6 inward currents at −60 mV were analyzed (Figure 2D). Again, we found that τ_H_ of the activation (Figure 2E) and deactivation kinetics (Figure 2F) were significantly faster using LEDs. τ_H_ of the activation kinetics was 16-fold lower using LED compared to the xenon lamp (12.3 ± 5.8 ms with LEDs and 195.0 ± 53.3 ms with xenon lamp) (Figure 2E) and τ_H_ of the deactivation kinetics was 4-fold faster (31.2 ± 22.2 ms with LEDs and 128.5 ± 8.8 ms with xenon lamp) (Figure 2F). The inactivation kinetics was again biphasic and significantly faster when using LEDs (Figures 2G–2I). τ_H_ of the fast inactivation was 8-fold lower using LED (0.4 ± 0.1 s with LEDs and 3.0 ± 1.2 s with xenon lamp) (Figure 2H) and τ_H_ of the slow inactivation was almost 3-fold lower (73.0 ± 48.0 s with LEDs and 187.5 ± 81.0 s with xenon lamp) (Figure 2I). These findings also support the notion that the intensity of the light source is crucial for sufficient photoswitching.

Furthermore, we determined the factors of current density increases between *trans* and *cis* isomer-induced currents. For OptoBI-1 we obtained activation factors of 11.6 ± 3.9 (median ±SD) for Xenon and 10.6 ± 2.9 for LED which were not significantly different (*p* = 0.99; Mann Whitney U test) for outward currents and 8.3 ± 4.0 for Xenon and 6.3 ± 5.7 for LED for inward currents which were also not significantly different (*p* = 0.83; Mann Whitney U test). For OptoDArG we obtained activation factors of 11.7 ± 4.8 for Xenon and 12.2 ± 9.9 for LED which were not significantly different (*p* = 0.86; Mann Whitney U test) for outward currents and 26.2 ± 11.2 for Xenon and 18.0 ± 20.7 for LED for inward currents which were also not significantly different (*p* = 0.81; Mann Whitney U test).

Moreover, we applied LED light with increasing light intensities of 25%, 50%, and 100% to TRPC6 expressing cells in the presence of OptoBI-1 (Figures S4E–S4H). Application of increasing light intensities resulted in significantly decreased half-live time constants (τ_H_) of activation, deactivation, and inactivation kinetics. These finding also suggest that the current kinetics strongly depend on the intensity of the light source.

Next, we determined the lightning power density of the two LEDs and of the xenon lamp in the beam path where the objective lens would normally be located. The wavelengths used for photoswitching were not identical but quite similar. At a wavelength of 445 ± 10 nm using the xenon lamp, we achieved a lightning power density of 0.59 ± 0.02 mW cm^−2^, and using the blue light LED with a peak wavelength of 442 nm we achieved 3.31 ± 0.10 mW cm^−2^. At a wavelength of 365 ± 10 nm using the xenon lamp, we measured 1.27 ± 0.13 mW cm^−2^, and using the UV light LED with a peak wavelength of 367 nm we achieved 7.96 ± 0.80 mW cm^−2^. Thus, the LEDs showed approximately 6-fold higher lightning power densities. Furthermore, a reduced LED light intensity of 25% resulted in lightning power density of 1.99 ± 0.20 mW cm^−2^ for UV light and 0.83 ± 0.02 mW cm^−2^ for blue light, and LED light intensity of 50% resulted in lightning power density of 3.98 ± 0.40 mW cm^−2^ for UV light and 1.66 ± 0.05 mW cm^−2^ for blue light. Our findings suggest that the light intensity of the xenon lamp is too low to induce sufficient and rapid *cis*-*trans* isomerization of OptoDArG and OptoBI-1. Therefore, low-intensity light sources are not suitable for fast photoswitching or for achieving fast current kinetics.

### Characterization of thermal relaxation, reversibility of photoswitching and photostationary states of OptoDArG, OptoBI-1, and PhoDAG

Unfortunately, the photoswitchable TRPC6 activators OptoDArG, OptoBI-1 and PhoDAG have not been extensively characterized until now. Therefore, we conducted a comparative characterization of the distinct properties of these compounds such as thermal relaxation, reversibility of photoswitching, and photostationary states (PSS) using UV-vis and NMR spectroscopy. Additionally, UV-vis spectroscopy was used to analyze the thermal relaxation rate which refers to the thermal transition from the *cis* to the *trans* configuration in the dark after illumination with 365 nm light. The half-life time constants for thermal relaxation (τ_H_) at a concentration of 50 μM are 425.82 ± 0.12 min for OptoDArG, 418.76 ± 0.13 min for OptoBI-1, and 396.34 ± 0.08 min for PhoDAG (Figure S5). These results indicate that the *cis* configuration exhibits high thermal stability, with complete relaxation to the *trans* configuration occurring after approximately 36 h at 37°C.

To determine the reversibility of photoswitching, the compounds were repeatedly illuminated with 365 nm and 460 nm light for 5 min each over a period of 110 min at 37°C (Figure S6). The absorption was measured at 340 nm. No changes in absorption were observed with respect to the respective illumination wavelengths, suggesting that photoswitching is fully reversible and that the compounds do not degrade during this period through e.g., photobleaching.

The PSS is the equilibrium state between the *trans* and *cis* configuration at a given illumination wavelength. To determine the PSS of the three photoswitchable compounds, we used UV-vis spectroscopy for near-quantitative analysis (Figure S7). We found that in the dark, after complete thermal relaxation, the highest percentage of the *trans* configuration is achieved at an absorption maximum (λ_max_) of 335 nm for OptoDArG, 336 nm for OptoBI-1, and 337 nm for PhoDAG. In order to obtain a PSS with the highest percentage of the *trans* configuration at λ_max_, the most appropriate wavelength was 435 nm for OptoDArG and 435 nm as well as 525 nm for OptoBI-1 and PhoDAG. Focusing on the illumination wavelength of 435 nm, which is closest to the wavelength used in patch-clamp measurements to induce the trans configuration, we found that OptoDArG achieves approximately 93% trans and consequently a calculated 7% *cis* configuration. OptoBI-1 achieves approximately 90% *trans* and 10% calculated *cis* configuration, while PhoDAG reaches around 92% *trans* and 8% *cis* configuration. Illumination at 365 nm results in the lowest absorption at λ_max_, suggesting that this wavelength is suitable for achieving a high percentage of the *cis* configuration.

For a more precise quantitative analysis of the PSS, we additionally used ^1^H-NMR spectroscopy (Figures S8–S10). We found that the freshly dissolved commercially obtained photoswitchable compounds (2.5 mM), without additional thermal relaxation, were almost entirely in the *trans* configuration (≥97%). Since OptoDArG contains two azobenzene moieties, illumination with 435 nm and 365 nm light results in a mixture of conformational states: entirely *trans* (*trans*+*trans*), mixed *trans* and *cis* (*trans*+*cis* or *cis*+*trans*), and entirely *cis* (*cis*+*cis*) configurations, depending on the isomerization state of the first and second fatty acid. The mixed *trans* and *cis* configurations were not discriminated. Illumination with 435 nm resulted in a ratio of 60% for the fully *trans*, 19% for the mixed, and 21% for the fully *cis* configuration. Illumination with 365 nm caused a ratio of 2% for the fully *trans*, 2% for the mixed, and 96% for the fully *cis* configuration. Illumination of OptoBI-1 with 435 nm caused a ratio of 78% *trans* and 22% *cis* configuration, and illumination with 365 nm results in a ratio of 4% *trans* and 96% *cis* configuration. Illumination of PhoDAG with 435 nm caused a ratio of 80% *trans* and 20% *cis* configuration, and illumination with 365 nm resulted in a ratio of 2% *trans* and 98% *cis* configuration.

### Photoswitching of OptoDArG, PhoDAG, and OptoBI-1 with LEDs elicits distinct TRPC6 current kinetics

Next, we tested the photoswitchable DAG derivative PhoDAG (200 μM)^13^^,^^14^^,^^15^ which contains only one photoswitchable azobenzene group as well as a stearoyl moiety. Hereby, we only used the more efficient LEDs as a light source. To test whether OptoDArG, OptoBI-1, and PhoDAG were applied in their maximally effective concentrations, we analyzed CD, current kinetics and NSC in the presence of increased concentrations of OptoDArG (200 μM) and OptoBI-1 (20 μM) (Figures S11–S14). Since 200 μM PhoDAG already represents the concentration which was maximally soluble in the bath solution, we reduced its concentration to 100 μM and repeated the measurements (Figures S15 and S16). We found that increasing the concentrations of OptoDArG or OptoBI-1 or decreasing the concentration of PhoDAG neither had an effect on the maximally induced CD nor on the current kinetics or on the NSC curve progression, suggesting that the chosen concentrations are already maximally effective concentrations.

**Figure 3:**
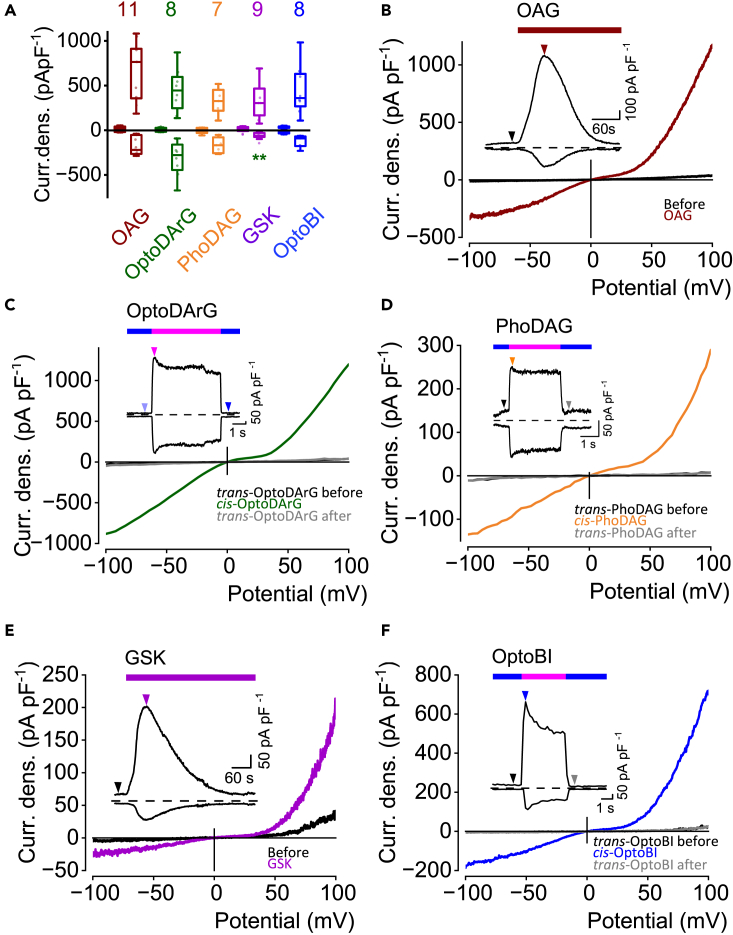
TRPC6 current densities elicited by photoswitchable and non-photoswitchable TRPC6 channel activators Electrophysiological whole-cell measurements of TRPC6 overexpressing HEK293T cells. (A) Summaries of current densities (“Curr. dens.”) at potentials of ±100 mV evoked by application 100 μM OAG, of 10 μM GSK 1702934A (“GSK”), of blue light and UV light using LEDs in the presence of OptoDArG, PhoDAG or OptoBI-1. First small boxplots represent current densities before application of OAG or GSK or induced by *trans-*OptoDArG, *trans-*PhoDAG or *trans-*OptoBI-1 and second boxplots maximal current densities induced by OAG, *cis-*OptoDArG, *cis-*PhoDAG, GSK or *cis-*OptoBI-1. Significant differences were calculated using Kruskal-Wallis test. Brown asterisks indicates significant differences compared to the OAG-induced current densities and green asterisks compared to OptoDArG-induced current densities (∗∗*p* < 0.01, ∗*p* < 0.05). Data are displayed as boxplots and interquartile ranges. Numbers over boxplots indicate number of measured cells. (B–F) Representative current density (“Curr. dens.”)-time courses at ±100 mV and current density-voltage relations. Bars indicate bath application of OAG (B, brown bar) or GSK (E, violet bar) or of blue light (C, D, F; blue bar) or UV light (C, D, F; magenta bar) using LEDs. The arrows indicate the time point at which the current density-voltage relations were selected.

Using 200 μM PhoDAG, photoswitching with 365 nm caused *cis*-PhoDAG-induced current density increases that were not significantly different from the *cis*-OptoDArG-induced current densities (Figures 3A and 3D) or the current densities induced by the membrane-permeable DAG analog OAG (100 μM) which was applied via perfusion with the bath solution (Figure 3B). To analyze whether *trans*-OptoDArG already binds to the channel, we used TRPC3 channels that possess a high constitutive activity compared to TRPC6 channels which exhibit almost no constitutive activity.^22^ These findings also suggest that blue LED light was quite efficient, producing a very high percentage of *trans* isomers, which led to channel inhibition. Interestingly, wash in of *trans*-OptoDArG via perfusion significantly inhibited the basal TRPC3 currents in a concentration-dependent way suggesting that *trans*-OptoDArG binds in the binding pocket thereby eliciting inhibitory effects (Figure S17A). In addition, we compared TRPC6 currents in the absence and presence of the *trans* isomers of OptoDArG, PhoDAG, and OptoBI-1 (Figure S17B). We did not observe any significant differences suggesting that the *trans* isomers have no additional effect on the already very low basal TRPC6 currents.

As a control, non-transfected HEK293T cells were analyzed in the presence of 30 μM OptoDArG, 10 μM OptoBI-1, and 200 μM PhoDAG. However, photoswitching did not elicit any significant current responses (Figure S18).

The non-lipidic TRPC6 activator GSK (10 μM) which was also applied via perfusion with bath solution (Figure 3E) and its derivative *cis*-OptoBI-1 (10 μM) (Figure 3E) caused similar TRPC6 current density increases. Only the *cis*-OptoDArG- and GSK-induced current densities at −100 mV were significantly different (Figure 3A).

**Figure 4:**
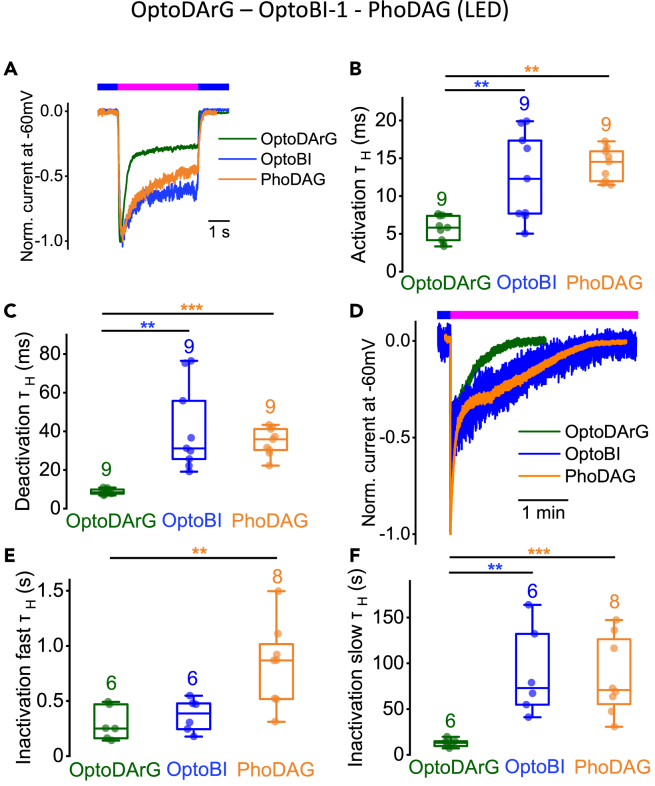
Photoswitching of OptoDArG, PhoDAG, and OptoBI-1 with LEDs results in distinct TRPC6 current kinetics Electrophysiological whole-cell measurements of TRPC6 overexpressing HEK293T cells. (A) Representative normalized time courses of TRPC6 currents at constant holding potential of −60 mV during photoswitching from blue light (blue bar) to UV light (magenta bar) and back to blue light (blue bar) using LEDs. (B and C) Summaries of half-life time constants (τ_H_) of the activation (B) and deactivation (C) kinetics (∗*p* < 0.05, ∗∗*p* < 0.01, Kruskal-Wallis test). (D) Representative normalized time courses of TRPC6 currents at constant holding potential of −60 mV induced by photoswitching from blue light to UV light using LEDs. The duration of light application (blue and magenta bar) is indicated. (E and F) Summaries of half-life time constants (τ_H_) of the fast (E) and slow (F) inactivation kinetics (∗*p* < 0.05, ∗∗*p* < 0.01; Kruskal-Wallis test). (B, C, E, and F) Data are displayed as boxplots and interquartile ranges. Numbers over boxplots indicate number of measured cells.

Comparing the normalized current time courses at a constant holding potential of-60 mV we observed differences in curve progressions of OptoDArG-, PhoDAG-, and OptoBI-1-induced currents (Figure 4A). The half-life time constant (τ_H_) of the PhoDAG-induced activation kinetics was 15.5 ± 4.5 ms, of the deactivation kinetics 35.2 ± 4.1 ms, of the fast inactivation kinetics 935 ± 383 ms and of the slow inactivation kinetics 86 ± 34 s τ_H_ of the activation and deactivation kinetics are significantly different between OptoDArG and OptoBI-1 and between OptoDArG and PhoDAG (Figures 4B and 4C). Comparing the fast inactivation kinetics, we observed significant differences between OptoDArG and PhoDAG, but not between OptoDArG and OptoBI-1 (Figures 4D and 4E). Comparing the slow inactivation kinetics, we found significant differences between OptoDArG and PhoDAG and between OptoDArG and OptoBI-1 (Figures 4D and 4F). These findings are pointing to the notion that different photoswitchable lipidic and non-lipidic TRPC6 channel activators induce different current kinetics. Moreover, different photoswitchable DAG derivatives cause distinct current kinetics, suggesting that each of these channel activators gives rise to different active channel states and distinct gating behaviors.

### Calculation of the normalized slope conductance reveals differences between photoswitchable and non-photoswitchable DAG derivatives

**Figure 5:**
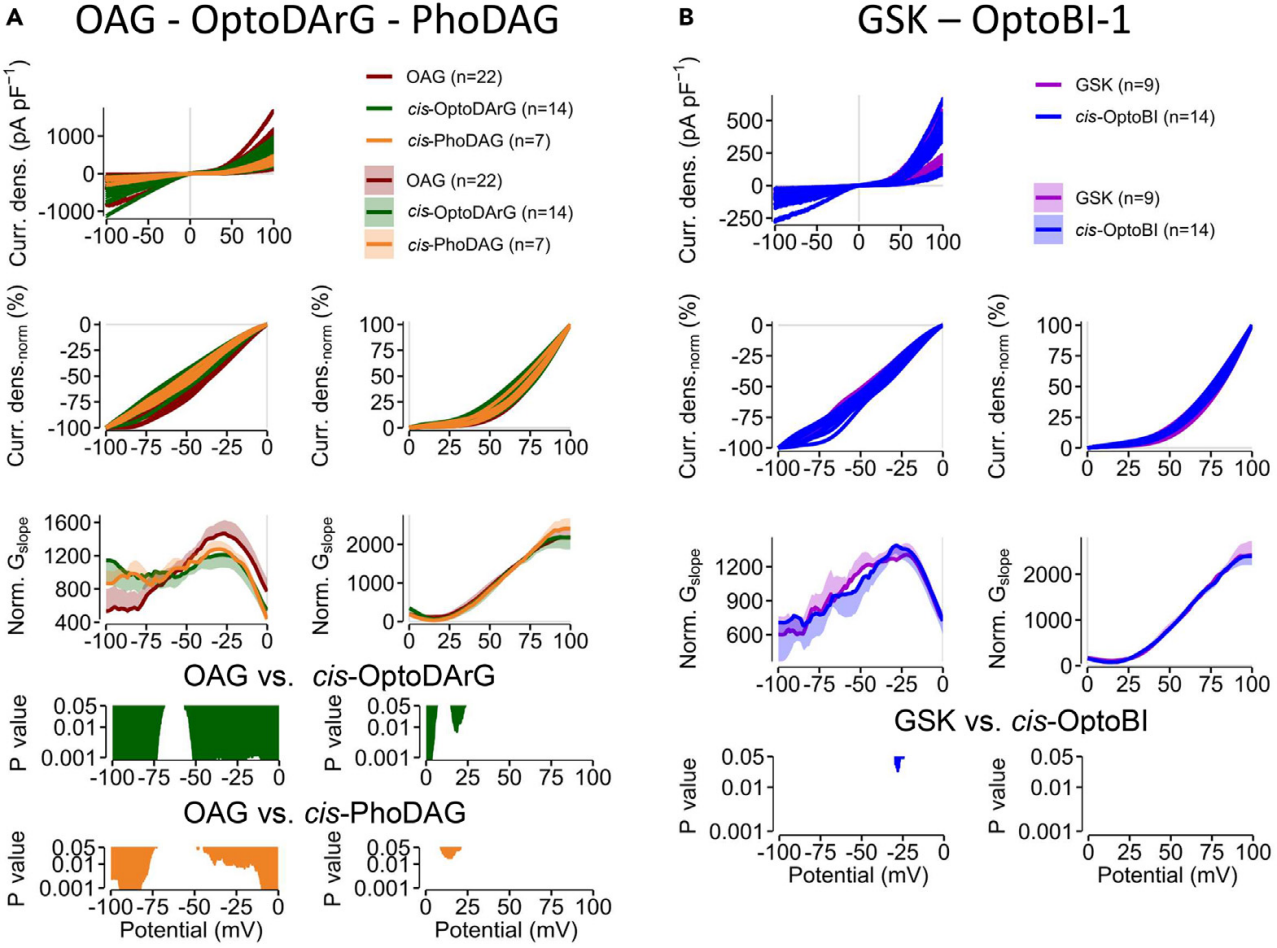
Calculation of the normalized slope conductance reveals differences between photoswitchable and non-photoswitchable TRPC6 activators Whole-cell measurements of TRPC6 overexpressing HEK293T cells. (A) All maximal current density-voltage relations (“Curr. dens.”) induced by OAG, *cis*-OptoDArG or *cis*-PhoDAG are displayed (previously). The current density-voltage relations were smoothed and normalized (“Curr. dens_norm_ [%]”). The calculated normalized slope conductance (NSC) (“Norm. G_slope_”) is displayed as mean ± SD. *p* values are calculated using Kruskal-Wallis test. (B) All maximal current densities (“Curr. dens.”) at potentials of ±100 mV induced by GSK or *cis*-OptoBI-1 are displayed (previously). The current density-voltage relations were smoothed and normalized (“Curr. dens_norm_ [%]”). The calculated NSC (“Norm. G_slope_”) is displayed as mean ± SD. *p* values are calculated using Mann-Whitney U test.

To quantify differences between the current-voltage relations elicited by the different TRPC6 channel activators and between photoswitchable and non-photoswitchable derivatives, we separately calculated the NSC of the current density-voltage relations at negative and positive potentials (Figures 5, S1, and S19). Comparing the DAG derivatives OAG, OptoDArG, and PhoDAG we observed significant differences particularly at negative potentials. These differences in the individual NSC curve progressions were most pronounced between OAG- and *cis*-OptoDArG-induced currents at potential ranges between −100 and −75 mV and −50 to 0 mV (Figure 5A). At positive potentials, the NSC curves mainly differed at potentials between 0 and +10 mV and around +20 mV. The NSC curve progressions of the *cis*-PhoDAG-induced currents compared to the OAG-induced currents also mainly differed at negative potentials. However, the differences were less pronounced compared to *cis*-OptoDArG and were only observed between −100 and −80 mV, −50 and 0 mV, and between around +15 mV. The NSC curve progression of the outward current was almost similar to OAG (Figure 5A). Altogether, different DAG derivatives elicit distinct NSC progressions which points to distinct active channel states. Comparison of the NSC of the maximal GSK-induced currents and the currents induced by its photoswitchable derivative *cis*-OptoBI-1 revealed only minimal differences at −25 mV (Figure 5B). These findings support the notion that in contrast to the different DAG derivatives, GSK and its photoswitchable derivative OptoBI-1 have the same activation mechanism and cause similar channel gating.

### Comparison of photoswitchable TRPC6 activators shows significant differences in the NSC progression

**Figure 6:**
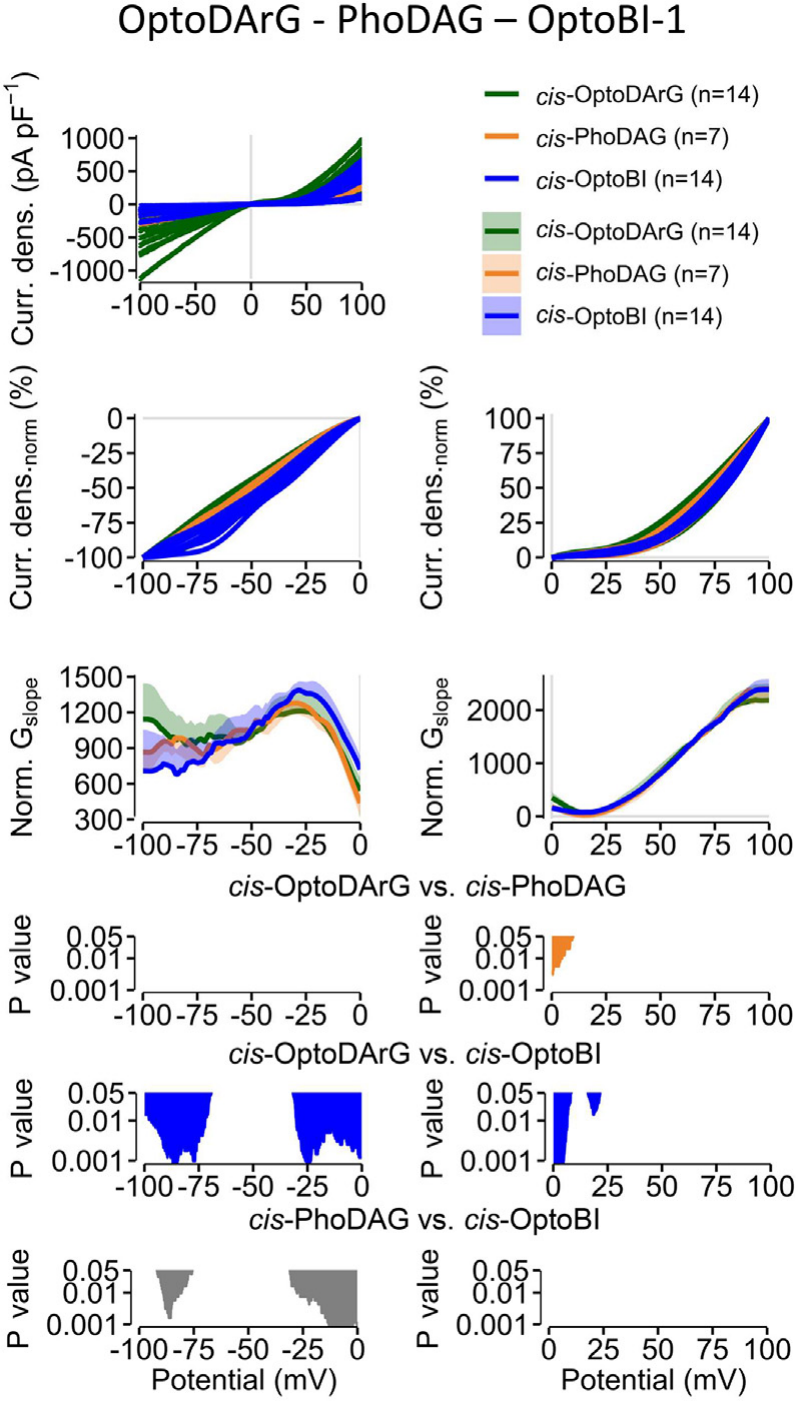
Comparison of photoswitchable TRPC6 activators shows significant differences in the NSC progression Whole-cell measurements of TRPC6 overexpressing HEK293T cells. All maximal current density-voltage relations (“Curr. dens.”) induced *cis*-OptoDArG, *cis*-PhoDAG or *cis*-OptoBI-1 are displayed (previously). The current density-voltage relations were smoothed and normalized (“Curr. dens_norm_ (%)”). The calculated NSC (“Norm. G_slope_”) is displayed as mean ± SD. *p* values are calculated using Kruskal-Wallis test.

When comparing the NSC progression of the lipidic and non-lipidic photoswitchable activators we observed that *cis*-OptoDArG- and *cis*-PhoDAG-induced currents show no significant differences at negative potentials, but at positive potentials between 0 and +12 mV significant differences are observed (Figure 6). The *cis* OptoBI-1-induced currents show distinctive differences at negative potentials between −100 and −70 mV and between −30 and 0 mV and at positive potentials between 0 and +10 mV and around +20 mV. Furthermore, the *cis*-OptoBI-1- and *cis*-PhoDAG-induced currents exhibit distinct NSC curve progressions at negative potentials between −80 and −75 mV and between −30 and 0 mV (Figure 6). These findings suggest that all three photoswitchable TRPC6 channel activators might induce distinct active channel states. However, the NSC of the currents induced by the photoswitchable DAG derivate are more similar than the currents induced by the photoswitchable GSK-derivative.

## Discussion

Photopharmacology is an emerging approach in medicine and life science that involves activation and deactivation of photoswitchable molecules with the spatiotemporal precision of light that might be useful for medical applications such as targeted drug delivery at a specific place and at a precise time.

The development of novel photoswitchable ion channel activators enabled the assessment of current kinetics in the whole-cell mode without interfering wash-in and wash-out effects that usually occur, when compounds are applied via bath solution.^15^ The determination of the current kinetics is of particular interest to characterize the pathophysiological role of TRPC6 channels. For example, current kinetics may be altered in patient mutations such as those in the gene coding for TRPC6 that are associated with FSGS. Interestingly, it was shown that some gain-of-function mutations that are co-expressed with wild-type TRPC6 exhibit a slower inactivation kinetics^23^ following receptor-activation. In addition, overexpression of TRPC7 channels in embryonic stem cell-derived cardiomyocytes enhanced the kinetics and frequency of intracellular calcium transients.^24^ In these cases, the use of light-switchable activators would be very useful to analyze the current kinetics more precisely, which might help to further elucidate the (patho-)physiological roles of distinct ion channels in health and disease.

The time span of the *trans* to *cis* isomerization of azobenzene moieties using a high-intensity mercury-xenon arc lamp for illumination was estimated to be in the two-digit picosecond range which was shown for small cyclic peptides containing an azobenzene moiety in the solvent DMSO by performing ultrafast IR spectroscopy.^25^^,^^26^ However, DMSO decelerates the transition time compared to water and the coupling of the azobenzene group to an 8 amino acid peptide chain also decelerates the transition time compared to a naked azobenzene group. However, the measured transition times vary strongly depending on the measurement method used.^27^ Using monochromatic LEDs or a lower intensity xenon lamp, it can be assumed that the power density is a thousand-fold lower compared to a high-intensity mercury-xenon arc lamp which might lead to a substantially slower isomerization time. However, even a 100,000-fold slower transition time of the azobenzene group, up to 1 μs, is sufficient and still faster than the channel gating, as the transition time of a single channel from the closed to the open state is about 10 μs.^28^ Hence, photoswitching of azobenzene groups would be sufficient for evaluating the gating behavior of single channels, and even more so for measurements of channel populations in the whole-cell configuration, because channel populations do not open synchronously but with a delay. Consequently, the time for isomerization is several orders of magnitude faster than the channel gating. It cannot be ruled out that the use of light with higher intensity such as laser light could even increase the current kinetics. However, by using LEDs, we have achieved a timescale that allows us to distinguish between the different current kinetics induced by various photoswitchable activators, enabling relative comparisons of channel kinetics. Nevertheless, the absolute kinetics might be faster.

Our findings suggest that when performing photopharmacology experiments, the selection of the light source for photoswitching is of utmost importance if current kinetics are to be analyzed. We found that xenon lamps are not powerful enough for fast and sufficient photoswitching (Figures 1 and 2) and that only high intensity light sources such as high-power LEDs are suitable. Furthermore, the switching time between the two excitation wavelengths was approximately 320 μs using a Polychrome V with a xenon lamp, and less than 5 μs using LEDs. This significant difference supports the use of LEDs, which is crucial for precise determination of current kinetics. Interestingly, the light power densities of the xenon lamp were about 6-times lower than those of the LED light at 100% intensity. Even at 25% light intensity, activation and deactivation kinetics achieved with LED light were still faster than with the xenon lamp. This suggests that not only light intensity but also the switching time between wavelengths is critical for fast and effective photoswitching (see Figure S4). However, if current kinetics are not a priority, low-intensity light sources can also be used, provided the light application is long enough to reach steady-state conditions. To determine maximal current densities, the complete channel activation should occur before significant inactivation effects interfere. Since ion channels open and close very quickly, particularly high light intensities and rapid light switching are required to obtain the most precise results. Nevertheless, there are instrument-based limitations when trying to conduct fast and precise photoswitching. Notably, since activation kinetics of GPCRs is much slower, ultrafast photoswitching of GPCR ligands is not necessary. Altogether, the current kinetics determined by photoswitching relies on the intensity of the light source as well as on the velocity of photoswitching.

Interestingly, we found that *trans*-OptoDArG causes inhibition of constitutive active TRPC3 channels indicating that the *trans* isoform already binds in the binding pocket of the channel. However, the inhibitory effect could not be detected using TRPC6 channels because of their lack of constitutive activity (Figure S17).

OptoDArG was previously shown to impact the membrane capacitance since *cis* isomerization results in reduced membrane thickness leading to an increased membrane capacitance.^29^ However, in our hands, photoswitching of OptoDArG or OptoBI-1 had no effect on the membrane capacitance of TRPC6 overexpressing HEK293T cells (Figure S20). In contrast to our experiments using 30 μM OptoDArG in living cells, Pfeffermann et al. (2021) employed a planar lipid bilayer composed by a membrane lipid and OptoDArG in a 9:1 ratio (90 M lipid and 10 M OptoDArG). In this experimental setup, OptoDArG was present in an exorbitantly higher concentration and photoswitching was performed using a low intensity xenon lamp which is not sufficient for fast photoswitching and might explain the slow kinetics determined in this study.

To summarize, performing whole-cell measurements and analyzing TRPC6 inward currents induced by photoswitching, we observed that photoswitchable TRPC6 channel activators are suitable for the reliable and reproducible determination of the activation, deactivation, and inactivation kinetics thereby allowing for a more detailed biophysical characterization of channel gating behavior.

Whole-cell measurements are commonly used to study ion channel activity in living cells. When analyzing whole-cell measurements, usually current densities and current-voltage relationships are used for biophysical characterization. Previously, we showed that the calculation of the NSC is beneficial for a detailed biophysical analysis of whole-cell currents.^21^ The NSC enables quantification of significant differences between current-voltage relations elicited by different stimuli or elicited by different ion channels.^21^ By calculating the NSC, voltage ranges can be identified that might be used for more detailed analysis e.g., on the single channel level. Applying this method, we observed differences between the NSC curve progressions induced by different channel modulators suggesting that different stimuli might induce distinct active channel states.^21^ Here, we demonstrate that combining the analysis of current kinetics enabled by photoswitchable compounds with the analysis of current-voltage relationships through NSC calculations allows for a more detailed biophysical characterization of whole-cell currents. This approach considers both current-voltage relations and current time courses, corroborating differences in gating behavior.

Comparison of the NSC progressions of the *cis*-OptoDArG and *cis*-PhoDAG evoked TRPC6 current voltage-relations revealed significant differences between 0 and +10 mV (see Figure 6). Furthermore, the current kinetics of the *cis*-OptoDArG and *cis*-PhoDAG induced TRPC6 currents at a holding potential of −60 mV revealed significant differences in the activation, deactivation, and inactivation kinetics (see Figure 4). In particular, the *cis*-PhoDAG induced current kinetics were significantly slower compared to the *cis*-OptoDArG induced currents kinetics. Thus, both analysis methods show significant differences suggesting that both photoswitchable DAG derivatives induce different active channel states thus reflecting different channel gating. Comparing the NSC of the current voltage-relations induced by the photoswitchable DAG derivatives to the non-photoswitchable DAG-derivative OAG that is washed in via the bath solution (Figure 5A), significant differences over a wide potential range were identified, additionally indicating that each DAG derivative elicits distinct gating behaviors. Interestingly, the differences in NSC and current kinetics were independent from the current density amplitudes (Figure 3A). Since all activators were applied in their maximally effective concentrations, we assume that all binding sites in the tetrameric TRPC6 channel are fully occupied. One piece of evidence for this is that the maximal current densities induced by OptoDArG, PhoDAG, and OptoBI-1 do not differ. However, all three channel activators cause differences in their current kinetics as well as in their NSC curve progressions. Under these conditions, differences in the NSC at a certain potential reflect differences in the product of the open probability (Po) and the single channel conductance at a certain potential (I_whole-cell_(Φ) = N x I_single channel_(Φ) x Po(Φ); and G_whole-cell_(Φ) = N x G_single channel_(Φ) x Po(Φ) with I: current; G: conductance; Po: open probability; N: number of channels; Φ: given potential). Given that the number of channels in the whole-cell configuration is constant and that all channels are occupied by the channel activator when using maximally effective concentrations, and given that the single channel conductance is usually constant (around 54 pS for TRPC6^22^) over the whole potential range, changes in the NSC are mainly due to changes in the Po meaning that the channel gating behavior is altered. Assuming comparable conditions (such as temperature, solutions, pipettes, parameters of whole-cell configuration, light intensities) and maximally effective concentrations, different activation, deactivation, and inactivation kinetics are pointing to different open channel states (O_1_, O_2_, O_3_ …), different inactivated channel states (I_1_, I_2_, I_3_ …) and different closed channel states (C_1_, C_2_, C_3_ …). Thus, we conclude that OptoDArG, PhoDAG, and OptoBI-1 induce their individual open, closed, and inactivated channel states. However, TRPC channel inactivation is still only poorly understood. We found no correlation between the inactivation kinetics and the current amplitudes (Figure S3) suggesting that the inactivation is independent of the current amplitudes. Presumably, current inactivation is an intrinsic feature of the channel that can be influenced extrinsically e.g., by phosphorylations.^30^^,^^31^ Further investigations are required to shed light on this phenomenon.

Altogether, the analysis of the current kinetics enables the determination of distinct open and closed states of the channel which was only possible before by analyzing single-channel currents. However, single channel measurements are technically more demanding and the single channel analysis is considerably more elaborate than whole-cell measurements.

As mentioned previously, DAG derivatives resulted in different current kinetics and differences in the NSC curve progression. Therefore, it can be speculated that TRPC6 channels can discriminate the different DAG derivatives and that the efficiency of each DAG derivative depends on the length and type of the fatty acid moieties. Although DAG is known as endogenous activator of TRPC channels^1^^,^^3^^,^^4^ the DAG molecule is not well defined since different fatty acid chains with different lengths and compositions including mono- or polyunsaturated fatty acids can naturally occur.^32^ The most common fatty acids chains found in the human plasma comprise oleic acid which is a mono-unsaturated omega-9 octadecenoic acid, palmitic acid which is a saturated hexadecenoic acid, stearic acid which is a saturated octadecanoic acid, linoleic acid which is a double-unsaturated omega-6 dienoic acid, myristic acid which is an unsaturated tetradecanoic acid, and arachidonic acid which is a quadruple-unsaturated omega-6 tetraenoic acid^32^ thus enabling a remarkable diversity of DAG molecules. At least 28 different 1,2-DAG molecules were identified.^32^ The DAG derivative OAG contains an oleic acid and a very short acetic acid chain. In contrast, PhoDAG contains a stearoyl acid chain and a photoswitchable fatty acid containing the photoswitchable azobenzene moiety, and OptoDArG contains two equal photoswitchable fatty acid with photoswitchable azobenzene moieties. Thus, the composition of the different DAG derivatives is variable making it likely that different derivatives establish distinct active channel states. Differences between *cis*-PhoDAG and *cis*-OptoDArG-induced currents have also been observed with TRPC3 currents^33^ and it was demonstrated that the deactivation kinetics of *cis*-OptoDArG-induced TRPC3, 6, and 7 currents differs.^33^ Nevertheless, in this study we show a detailed analysis of the activation, deactivation, and inactivation kinetics for the first time and our results support the notion that different channel activators cause distinct active channel states with different gating behaviors.

Although recent advances in cryo-EM revealed a potential binding site for DAG in the pore region of the TRPC5 channel which is located between two adjacent TRPC5 protein subunits,^34^ the channel regulation by DAG is still not completely understood, since the channel resides in its inactive state with a closed channel pore.^35^ However, photoswitchable DAG-derivatives may help to elucidate the lipid regulation in more detail,^12^^,^^33^^,^^36^ which was demonstrated by analyzing TRPC3 channels. Hereby, a point mutation in TRPC3 (G652A) was identified which leads to enhanced sensitivity to *cis*-OptoDArG but shows reduced sensitivity to *cis*-PhoDAG.^12^ Nevertheless, the lipid regulation of TRPC channels is still not fully understood and needs further investigations.

Photoswitchable compounds are not only effective in the heterologous overexpression system, but they can also be employed to study endogenously expressed channels in primary cells, tissues or organs. For example, PhoDAG and OptoDArG were effective on mouse vomeronasal sensory neurons and olfactory type B cells,^14^^,^^15^ and on mouse pancreatic islets and hippocampal neurons.^13^ In addition, OptoBI-1 was shown to be effective on human umbilical vein endothelial cells, and on freshly isolated murine hippocampal neurons.^16^ However, for *in vivo* applications or for analyzing tissue slices or organs, UV light that is used for photoswitching has some disadvantages since it is phototoxic and has a low permeation depth into skin and tissue. Fortunately, the first red light-switchable channel modulators that are activated by harmless red light are already available such as the red-light-switchable TRPV1 channel activator red-AzCA-4.^19^ This approach might help to overcome these obstacles.

Altogether, photoswitchable channel modulators allow for an extended biophysical analysis of whole-cell currents. Analysis of the current kinetics and the NSC in combination with structural information derived by cryo-EM might be beneficial to specify interactions between ligands and ion channels and might provide deeper insight into the transduction mechanisms leading to channel gating. In particular, 3D structures of ion channels in combination with photoswitchable activators in their *cis* and *trans* configuration would be important to shed light on the distinct conformational changes of the channel that occur during photoswitching. These findings might be advantageous for the determination of structure-function relations and might enable the design of novel potent channel modulators for the treatment of TRPC channel-dependent diseases. Moreover, the detailed structure-function relations might even allow for synthetic biology and the development of artificial ion channels with distinct functions.

### Limitations of the study

The following caveats should be taken into account when analyzing the current kinetics: The use of the appropriate light source is crucial for the reliable determination of the channel kinetics. In particular, the light intensity is of utmost importance for fast and complete photoswitching which is the prerequisite for determination of channel kinetics. Hereby, the switching time between the excitation wavelengths should be as short as possible.

The experiments were all performed in the heterologous overexpression system in HEK293T cells and endogenously expressed TRPC6 channels or TRPC6 channels in other heterologous overexpression systems have not been analyzed.

Furthermore, using sub-maximally effective concentrations of the photopharmaceuticals will decelerate the current kinetics. Thus reliable determination of the current kinetics can only be achieved when using photopharmaceuticals in their maximally effective concentrations.

The Nyquist-Shannon sampling theorem is an essential principle that must be observed.

## Resource availability

### Lead contact

Further information and requests for resources and R and MATLAB codes should be directed to and will be fulfilled by the lead contact, Prof. Dr. Michael Mederos y Schnitzler (mederos@lrz.uni-muenchen.de).

### Materials availability

This study did not generate new unique reagents.

### Data and code availability

• All data reported in the paper will be shared by the lead contact upon request.
• This paper reports original code for R and MATLAB which is available as supplemental data.
• Any additional information required to reanalyze the data reported in this paper is available from the lead contact upon request.

## Acknowledgments

We thank Laura Danner and Sebastian Pöll for excellent technical support. In addition, we thank Jürgen Aust and Michael Etterer for manufacturing of pipette holder and of measurement chamber and we thank Christoph Brenker for providing the connection unit based on the IC-HG30. This work was supported by the 10.13039/501100001659German Research Foundation (Deutsche Forschungsgemeinschaft) project no. ME 2456/4-1 and TRR-152 project no. P26 and by a Liebig fellowship from the Fonds der chemischen Industrie (FCI).

## Author contributions

M.MyS. and U.S. designed experiments and supervised the study. M.K. performed patch-clamp experiments. D.B.K. designed and supervised the chemical analysis. K.L. conducted chemical analysis and analyzed the chemical data. M.K., C.H., and M.MyS. analyzed the patch-clamp data. U.S. and M.MyS. wrote the manuscript. T.G. and D.B.K. revised the manuscript. C.H. prepared LED control and fit routine with MATLAB R2023a. M.K., M.MyS., U.S. and K.L. prepared the figures. All authors reviewed the manuscript.

## Declaration of interests

The authors declare no competing interests.

## STAR★Methods

### Key resources table

**Table:** 

REAGENT or RESOURCE	SOURCE	IDENTIFIER
**Chemicals, peptides, and recombinant proteins**		

2-Acetyl-1-oleoyl-*sn*-glycerin	Sigma-Aldrich	Cat. No. 495414
PhoDAG	Sigma-Aldrich	Cat. No. 870621P
Poly-L-Lysine	Sigma-Aldrich	Cat. No. P-1524
Bovine Serum Albumin (BSA)	Sigma-Aldrich	Cat. No. A7030
OptoBI-1	Bio-Techne	Cat. No. 7013
GSK 1702934A (GSK)	Tocris	Cat. No. 6508
OptoDArG	Aobious	Cat. No. AOB31427
DMSO, anhydrous	Sigma-Aldrich	Cat. No. 472301
Genejuice	Merck	Cat. No. 70967
Earl’s MEM	Sigma-Aldrich	Cat. No. M4655
Penicillin-Streptomycin	Sigma-Aldrich	Cat. No. P4333
Fetal calf serum (FSC)	Gibco	Cat. No. 10270106
HEPES (2-[4-(2-hydroxyethyl)-1-piperazinyl]-ethanesulfonic acid)	Sigma-Aldrich	Cat. No. H0887
BAPTA (1,2-bis(2-aminophenoxy)ethane-N,N,N′,N′-tetraacetic acid)	Sigma-Aldrich	Cat. No. A4926
CsCl	Sigma-Aldrich	Cat. No. 203025
Na_3_-GTP	Sigma-Aldrich	Cat. No. G8877
CsOH	Sigma-Aldrich	Cat. No. C8518
NaCl	Sigma-Aldrich	Cat. No. N31434
Glucose	Sigma-Aldrich	Cat. No. G7528
MgCl_2_	Sigma-Aldrich	Cat. No. M2670
CaCl_2_ solution, 1 M	Sigma-Aldrich	Cat. No. 21115
Deuterated DMSO ((CD_3_)_2_SO)	Eurisotop	Cat. No. D010F
Deuterium oxide (D_2_O)	Eurisotop	Cat. No. D214F

**Experimental models: Cell lines**		
Human embryonic kidney (HEK293T) cells	Leibniz-Institute DSMZ	DSMZ No. ACC 635

**Recombinant DNA**		

pIRES2-EGFP expression vector	Clontech	N/A
mouse TRPC6	(Zhu et al., 1996)^37^	GenBank: NM_013838
human TRPC3	(Boulay et al., 1997)^38^	GenBank: NM_001130698

**Software and algorithms**		

Patchmaster version v2x90.5	Heka	N/A
Origin 2023	OriginLab	N/A
MATLAB R2023a	MathWorks Inc	N/A
R 4.2.3	R Core Team	N/A
MestReNova v.10.0.1–14719	Mestrelab Research	N/A
Cary WinUV	Agilent	N/A

**Other**		

Glass cover slips 40 mm diameter, thickness 1	Karl-Hecht	Cat. No. 92100101180
LED M365LP1	Thorlabs	N/A
LED M450LP2	Thorlabs	N/A
Optical band-pass filter 360/23 BrightLine HC	AHF	Cat. No. F39-363
Optical band-pass filter 433/24 BrightLine HC	AHF	Cat. No. F37-433
Dichroic beamsplitter HC 376	AHF	Cat. No. F38-376
Dichroic beamsplitter HC 458	AHF	Cat. No. F38-458
Monochromator with xenon short-arc lamp	Till Photonics	Polychrome V
Inverted microscope IX 70	Olympus	N/A
Dichroic beamsplitter H 488 LPXR superflat Vers 2 with Emission filter 525/50 BrightLine HC for microscope	AHF	Cat. No. F48–487 and F37-516
40× oil UV-transmissive apochromatic objective UApo *N* 340	Evident	N/A
Amplifier EPC 10 USB	Heka	N/A
Energy meter Nova II with sensor PD300	Ophir	N/A
self-made control unit with IC-HG30 laser switches mounted on EVAL HG1D evaluation boards	iC-Haus	N/A
EVAL HG1D evaluation boards	iC-Haus	N/A
microcontroller board Arduino Mega 2560	Arduino SA	N/A
borosilicate glass	Science Products	Cat. No. GB150TF-8P
NMR spectrometer Avance III HD 500 MHz	Bruker	N/A
BioSpin with CryoProbe^TM^ Prodigy	Bruker	N/A
Cary 60 UV-Vis spectrophotometer	Agilent	Cat. No. G6860A
High Precision Cell (quartz glass, 10.00 mm light path)	Hellma Analytics	Cat. No. 104-QS
Illumination system pE-4000	CoolLED	Cat. No. pE-4000

### Experimental model and study participant in details

#### Cell lines

In this study, we used human embryonic kidney (HEK293T) cells (from Leibniz-Institute DSMZ, Braunschweig, Germany, T293, DSMZ no. ACC 635).

### Method details

#### Materials

2-Acetyl-1-oleoyl-*sn*-glycerin (OAG; Cat. No. 495414), PhoDAG (Cat. No. 870621P), Poly-L-Lysine (Cat. No. P-1524), and Bovine Serum Albumin (BSA; Cat. No. A7030) were purchased from Sigma-Aldrich (Taufkirchen, Germany). OptoDArG was purchased from Aobious (Gloucester, USA; Cat. No. AOB31427), OptoBI-1 was purchased from Bio-Techne (Minneapolis, USA; Cat. No. 7013) and GSK 1702934A (GSK) was purchased from Tocris (Bristol, UK; Cat. No. 6508). OptoDArG and PhoDAG were solved in anhydrous DMSO to 50 mM, OptoBI-1 was solved in anhydrous DMSO to 10 mM, OAG and GSK were solved in anhydrous DMSO to 100 mM. PhoDAG and OptoDArG stock solutions were stored in aliquots at −20°C for maximal 4 weeks. Deuterated DMSO ((CD_3_)_2_SO) and deuterium oxide (D_2_O) were purchased from Eurisotop. Purchased solvents in HPLC- and analytical-grade quality were used without further purification.

#### Nuclear magnetic resonance (NMR) spectroscopy

To quantitatively determine the photostationary states (PSS) of OptoDArG, OptoBI-1 and PhoDAG, we analyzed NMR-spectra that were acquired with the spectrometer Avance III HD 500 MHz (Bruker) BioSpin with CryoProbe Prodigy. Deuterated DMSO ((CD_3_)_2_SO) and deuterium oxide (D_2_O) were used as internal references. Spin multiplicities are described as follows: s (singlet), d (doublet), t (triplet), m (multiplet) or a combination thereof. Spectra analysis was conducted with the software MestReNova v.10.0.1–14719. The experiments were conducted using a 2.5 mM solution of OptoDArG, OptoBI-1 and PhoDAG in (CD_3_)_2_SO:D_2_O (9:1) at 22°C.

#### UV-Vis spectroscopy

To determine the PSS nearly quantitatively, as well as the reversibility of photoswitching and the thermal relaxation of OptoDArG, OptoBI-1, and PhoDAG, UV-Vis spectroscopy was applied. UV-Vis spectra were recorded on a Cary 60 UV-Vis spectrophotometer (Agilent) and as cuvette a High Precision Cell (quartz glass, 10.00 mm light path; Hellma Analytics) was used. Analysis was conducted with the Cary win UV/Scan.exe software. Illumination was provided by the illumination system pE-4000 from CoolLED. The optical power output of the LEDs according to CoolLED is 10 mW/mm^2^ for 525 nm, 15 mW/mm^2^ for 500 nm, 50 mW/mm^2^ for 490 nm, 50 mW/mm^2^ for 470 nm, 50 mW/mm^2^ for 460 nm, 40 mW/mm^2^ for 435 nm, 50 mW/mm^2^ for 405 nm, 50 mW/mm^2^ for 385 nm and 50 mW/mm^2^ for 365 nm. The experiments were conducted using a 50 μM solution of OptoDArG, OptoBI-1 and PhoDAG in (CD_3_)_2_SO:D_2_O (9:1) at 37°C.

#### Cell culture and transfection

HEK293T cells were kept in Earl’s MEM (Sigma-Aldrich) with 100 units/mL penicillin and 100 μg/mL streptomycin supplemented with 10% (v/v) FCS (Gibco, Life Technologies, Carlsbad, USA). All cells were held at 37°C in a humified atmosphere with 5% CO_2_. HEK293T cells were transfected with 2 μg cDNA coding for mouse TRPC6 (GenBank: NM_013838)^37^ or with human TRPC3 (GenBank: NM_001130698)^38^ using Genejuice reagent (Sigma-Aldrich) according to the manufacturer’s instructions. The cDNA was in pIRES2-EGFP expression vector (Clontech, Palo Alto, CA). Transfected HEK293T cells were seeded onto poly-L-Lysine-coated glass cover slips (diameter 30 mm, thickness 1, Karl Hecht, Sondheim, Germany) 1 h before patch clamp measurements.

#### Light stimulation

For light stimulation, two LEDs from Thorlabs (M365LP1 with a peak wavelength of 367 nm and M450LP2 with a peak wavelength of 442 nm; Bergkirchen, Germany) with optical band pass filters from AHF (360/23 BrightLine HC and 433/24 BrightLine HC; Tübingen, Germany) were used that were connected over dichroic beamsplitters from AHF (HC 376 and HC 458, respectively) and that elicited effective light with wavelengths between 346 and 371 nm and between 418 and 447 nm. Comparable wavelengths of 365 ± 10 nm and 445 ± 10 nm were applied using the monochromator Polychrome V with a xenon short-arc lamp (Till Photonics, Planegg, Germany) with maximal power which allowed for a switching time between the excitation wavelengths from 445 to 365 nm and vice versa of about 320 μs. An Olympus IX70 microscope was used with dichroic beamsplitter (H 488 LPXR superflat Vers. 2) combined with the emission filter (525/50 BrightLine HC) from AHF and the 40× oil UV-transmissive apochromatic objective (UApo *N* 340; Evident, Hamburg, Germany). The light intensities were determined at the position in the beam path where the objective lens would normally be located using the laser power or energy meter Nova II with sensor PD300 (Ophir; Jerusalem, Israel). The LEDs were operated by a self-made control unit with IC-HG30 laser switches mounted on EVAL HG1D evaluation boards from iC-Haus (Bodenheim, Germany) connected to the microcontroller board Arduino Mega 2560 (Arduino SA, Chiasso, Switzerland). A self-written MATLAB app (R2023a; MathWorks Inc.; Natick, Massachusetts, USA) served as user-interface driving the microcontroller through serial communication. The effective time of switching between the two LEDs was not more than 5 μs. The LEDs operated with intensity levels of an 8-bit sigmoidal scale.

#### Patch-clamp recordings

For patch-clamp measurements, OptoBI-1, OptoDArG, and PhoDAG stock solutions were heated for short time thermal relaxation to 40°C for 10 min and diluted in standard bath solution containing 140 mM NaCl, 5 mM CsCl, 1 mM MgCl_2_, 2 mM CaCl_2_, 10 mM glucose, 10 mM HEPES (pH 7.4 with NaOH) resulting in an osmolarity of 295–302 mOsm⋅kg^−1^ to the final standard concentration of 10 μM for OptoBI-1, 30 μM for OptoDArG, and 200 μM for PhoDAG, just before the measurements. Some indicated measurements were conducted with 200 μM OptoDArG, 100 μM PhoDAG, and 30 μM OptoBI-1. After dilution, the OptoBI-1 solution was used within 1 h. The solutions were illuminated with blue light prior to application to the cells. Conventional whole-cell patch-clamp recordings were carried out at room temperature (23°C) 48 h after transfection. Photoswitching either with LEDs or with xenon lamp had no temperature effects. The mTRPC6 over-expressing HEK293T cells on coverslips were incubated with 30 μM OptoDArG or 200 μM PhoDAG solution for 20 to 30 min at room temperature prior to the measurements. The 10 μM OptoBI-1 solution did not have to be incubated. In the case of OAG and GSK measurements, the cells were superfused with 100 μM OAG plus 0.1% fatty-acid-free BSA and 10 μM GSK. OAG, GSK, OptoDArG, PhoDAG, and OptoBI-1 were used in their maximally effective concentrations. The standard pipette solution contained 120 mM CsCl, 9.4 mM NaCl, 0.2 mM Na_3_-GTP, 1 mM MgCl_2_, 3.949 mM CaCl_2_, 10 mM BAPTA (100 nM free Ca^2+^), and 10 mM HEPES (pH 7.2 with CsOH), resulting in an osmolality of 294 mOsm kg^−1^. The liquid junction potential of +4.0 mV was calculated by JPCalcWin 1.01 (University of New South Wales, Sydney, Australia) and was corrected before the measurements. Data were collected with an EPC10 patch clamp amplifier (HEKA Elektronik, Lambrecht, Germany) using the Patchmaster software. Transfected cells were selected by application of light of the wavelength 445 nm to detect their green fluorescent protein EGFP.

For the determination of current density-voltage relationships and normalized slope conductances, repetitive voltage-upramps from −100 to +100 mV were applied. Current-densities analysis and current density-time courses were determined at the holding potential of +100 and −100 mV. For OAG and GSK measurements, a stimulation protocol with a frequency of 2 Hz was applied starting with a holding potential of −100 mV for 50 ms, followed by a voltage upramp from −100 to +100 mV for 400 ms and a holding potential of +100 mV for 50 ms. After each stimulation protocol a capacitance compensation was automatically conducted. To obtain current density-time courses of OptoDArG-, PhoDAG- and OptoBI-1-induced currents, a stimulation protocol with faster voltage upramps was applied with a frequency of 50 Hz starting with a holding potential of −100 mV that was applied for 7 ms, followed by a voltage upramp from −100 to +100 mV for 10 ms and a holding potential of +100 mV for 3 ms to detect fast changes of whole-cell currents. Hereby, data were acquired at a frequency of 5 kHz after filtering at 2.5 kHz. Kinetics of channel activation, deactivation and inactivation were obtained at a holding potential of −60 mV with a sampling frequency of 2 kHz. Patch pipettes were made of borosilicate glass from Science Products (Hofheim, Germany; Cat. No. GB150TF-8P) and had resistances of 1.8–3.2 MΩ.

#### Normalized slope conductance

The normalized slope conductances (NSCs) were calculated using current voltage relations that were selected at maximal activator-induced current amplitudes or using current-voltage relations before application of the activator or in the presence of blue light before application of UV light. Only current-voltage relations determined by application of a stimulation protocol with a frequency of 2 Hz starting with a holding potential of −100 mV for 50 ms, followed by a voltage upramp from −100 to +100 mV for 400 ms and a holding potential of +100 mV for 50 ms were used for NSC calculation. The calculation was done as described in Hermann et al.^21^ with the smoothing cubic spline fit and subsequent normalization of current-density voltage relations. Furthermore, smoothed and normalized inward and outward current densities and corresponding NSCs with *p* values were displayed separately.

#### Fit routine

To determine the half-life time constant of activation, deactivation and inactivation (τ_H_), currents determined at the holding potential of −60 mV were normalized and the time was shifted to zero for fitting with MATLAB R2023a. For τ_H_ of activation, the median of the current before activation was set to zero and ca. 30% of the maximal activated current was set to +1 to exclude the emerging interfering inactivation. For τ_H_ of deactivation, the median of the current before deactivation was set to +1 and the median of fully-deactivated current was set to zero. For τ_H_ of inactivation the peak current was set to +1 and the median of the steady-state current after inactivation was set to zero. Activation and deactivation were fitted by a mono-exponential function (Equations 1 and 2). Inactivation was fitted by a bi-exponential function (Equation 3). Fit optimization was performed with a quadratic error function calculating the summed square of residuals (SSE) (Equations 4 and 5) using the *fminsearch* function and the options described in Table 1.

(Equation 1) $$f_{activation}\left(t\right)=a*e^{\frac{\ln\;2*t}{\tau_{H}}}+c$$

Equation 1*: Mono-exponential fit function f*_*activation*_*dependent on time (t), initial quantity (a), time constant (*τ_H_*) and offset (c)*.

(Equation 2) $$f_{deactivation}\left(t\right)=a*e^{-\frac{\ln\;2*t}{\tau_{H}}}+c$$

Equation 2*: Mono-exponential fit function f*_*deactivation*_*dependent on time (t), initial quantity (a), time constant (*τ_H_*) and offset (c)*.

(Equation 3) $$f_{inactivation}\left(t\right)=a_{1}*e^{-\frac{\ln\;2*t}{\tau_{H1}}}+a_{2}*e^{-\frac{\ln\;2*t}{\tau_{H2}}}+c$$

Equation 3*: Bi-exponential fit function f*_*inactivation*_*dependent on time (t), initial quantities (a*_*1*_*; a*_*2*_*), time constants (*τ_H1_, τ_H2_*) and offset (c)*.

(Equation 4) $$SSE=\Sigma_{t}{\left[y_{t}-f\left(a,c,\tau_{H},t\right)\right]}^{2}$$

Equation 4*: Quadratic error function for mono-exponential fits using the summed square of residuals (SSE) with y*_*t*_*being the current at time t and f(a, c,* τ_H_*, t) the result either of* Equation 1 *or* Equation 2 *at time t*.

(Equation 5) $$SSE=\Sigma_{t}{\left[y_{t}-f\left(a_{1},a_{2},c,\tau_{H1},\tau_{H2},t\right)\right]}^{2}$$

Equation 5*: Quadratic error function for bi-exponential fits using the summed square of residuals (SSE) with y*_*t*_*being the current at time t and f(a, c,* τ_H1,_ τ_H2_*, t) the result of* Equation 3 *at time t*.

### Quantification and statistical analysis

Statistical analyses were conducted in R 4.2.3 and in Origin 2023 (OriginLabs, Northhampton, USA). No statistical methods were used to predetermine sample size. The *p* values were calculated by using an unpaired Mann-Whitney-U, a Wilcoxon matched pairs signed rank, one sample Wilcoxon test or Kruskal-Wallis or Friedman test with Dunn’s multiple comparison post hoc analysis. A *p* value less than 0.05 was considered significant for all analysiKey rs. ∗*p* < 0.05; ∗∗*p* < 0.01; ∗∗∗*p* < 0.001. Boxplots display the median and the interquartile range. Values of half-life time constants (τ_H_) are indicated as mean ± standard deviation (SD). The statistical tests used for statistical analysis can be found in the figure legends.

## Supplemental information

Document S1. Figures S1–S20

Data S1. MATLAB und R Code
