# Supplementary material for: Photoswitchable TRPC6 channel activators evoke distinct channel kinetics reflecting different gating behaviors
Source: iScience. 2024 Sep 20;27(10):111008. doi: 10.1016/j.isci.2024.111008 (PMC11472632; doi:10.1016/j.isci.2024.111008)
Supplement: Document S1. Figures S1–S20 [file mmc1.pdf]

iScience, Volume *27*

Supplemental information

Photoswitchable TRPC6 channel activators evoke distinct channel kinetics reflecting different gating behaviors

Maximilian Keck, Christian Hermann, Kyra Lützel, Thomas Gudermann, David B. Konrad, Michael Mederos y Schnitzler, and Ursula Storch

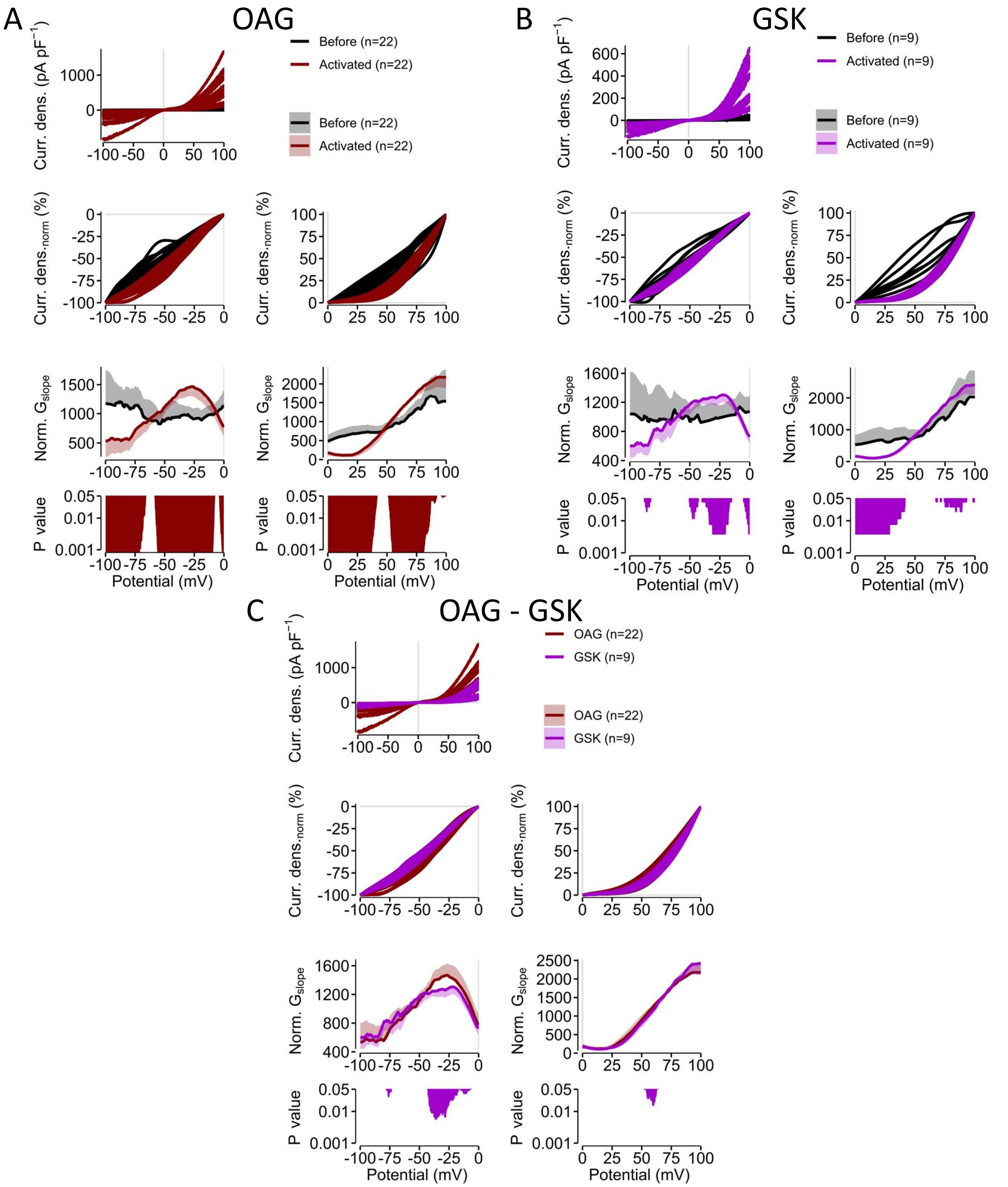

Supplemental Figure 1: Normalized slope conductance of TRPC6 currents before and during application of OAG or GSK. Whole-cell measurements of TRPC6 overexpressing HEK293T cells. (**a, b**) All current density-voltage relations ('Curr. dens.') before and during application of OAG (**a**) or GSK (**b**) are displayed (above). The current density-voltage relations were smoothed and normalized ('Curr. $dens_{norm}$ (%)'). The calculated normalized slope conductance (NSC) ('Norm. G_{slope}') is displayed as mean ± SD. P values are calculated using Wilcoxon matched signed-rank test. (**c**) Maximal current density-voltage relations ('Curr. dens.') during application of OAG or GSK are displayed (above). The current density-voltage relations were smoothed and normalized ('Curr. $dens_{norm}$ (%)'). The calculated NSC ('Norm. G_{slope}') is displayed as mean ± SD. P values are calculated using Mann-Whitney U test. **Related to Figures 3 and 5.**

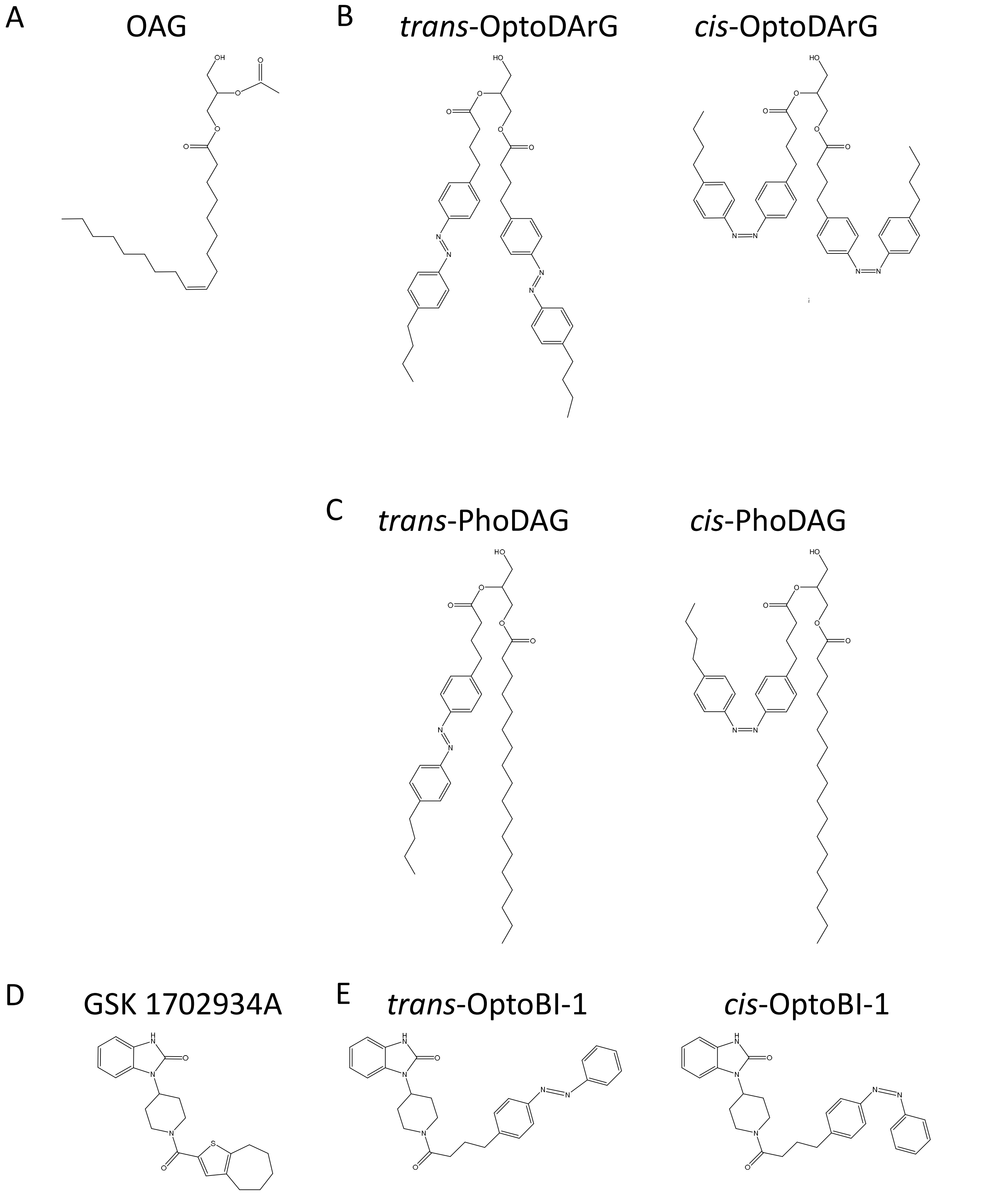

Supplemental Figure 2: Chemical structures of TRPC6 activators. Depiction of the chemical structures of lipidic (**a**-**c**) and non-lipidic TRPC6 activators used in the study (**d**, **e**). **Related to Figures 1-6.**

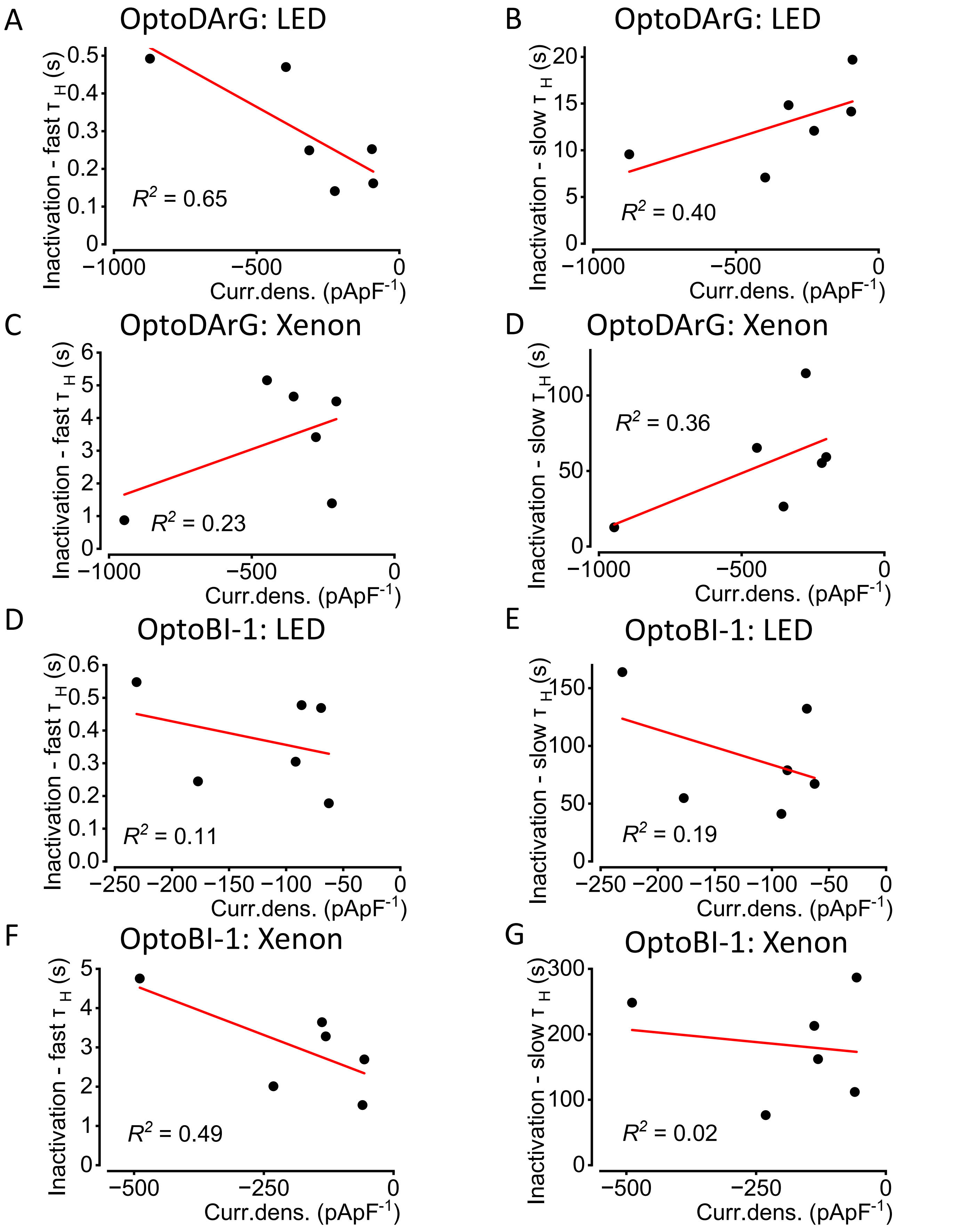

Supplemental Figure 3: Inactivation kinetics of TRPC6 currents induced by OptoDArG or OptoBI-1 are independent of current amplitudes. Correlation of current density amplitudes at -60 mV with fast and slow inactivation kinetics in the presence of OptoDArG (**a**-**d**) or OptoBI (**d**-**g**) with illumination using LEDs (**a**, **b**, **d**, **e**) or xenon lamp (**c**, **d**, **f**, **g**). **Related to Figures 1 and 2.**

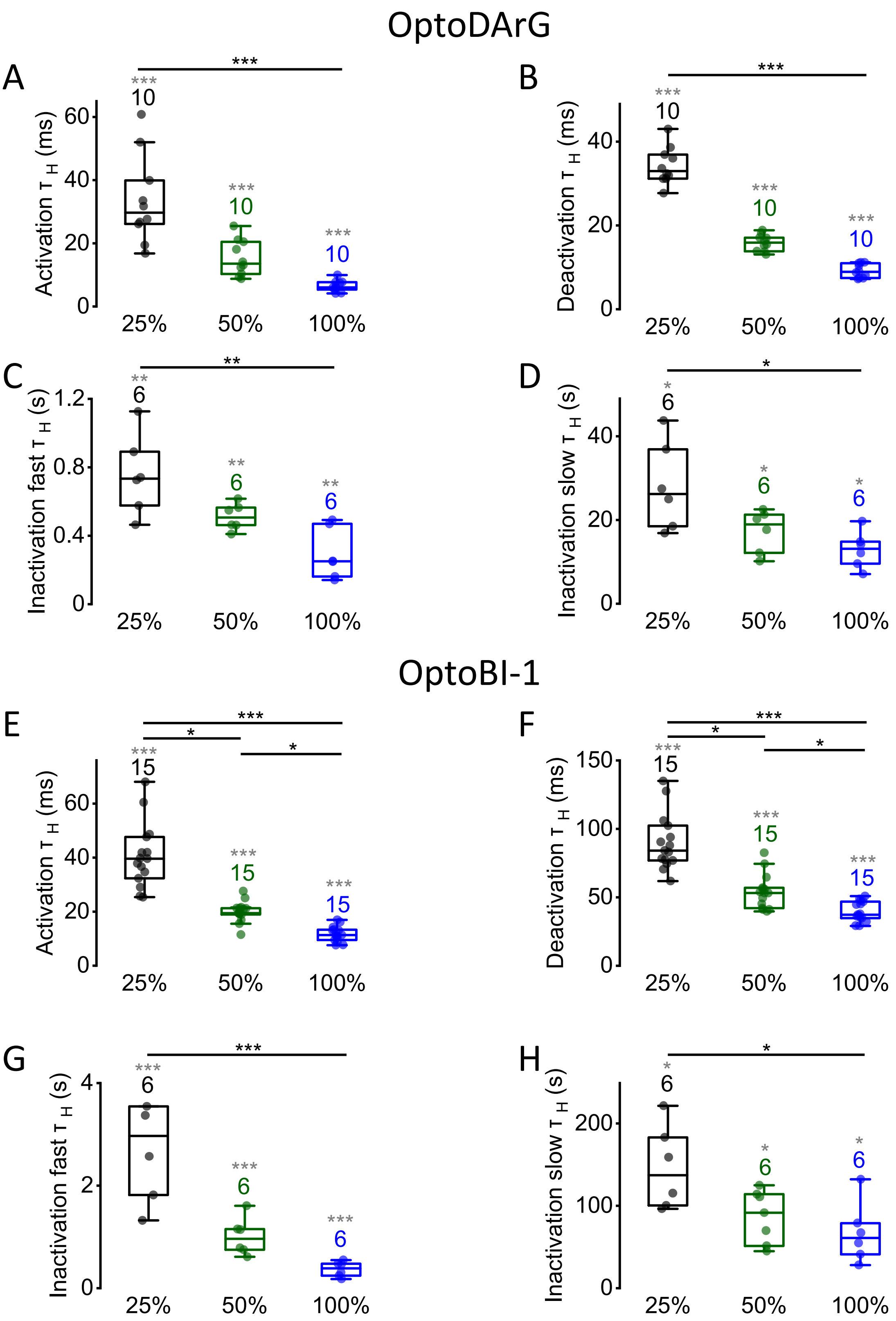

Supplemental Figure 4: Lower LED light intensities result in slower current kinetics. Whole-cell measurements of TRPC6 overexpressing HEK293T cells in the presence of 30 µM OptoDArG (**a**-**d**) or of 10 µM OptoBI-1 (**e**-**h**) during application of LED light with light intensities of 25%, 50% or 100%. Summaries of half-life time constants (τ_H) of the activation (**a**, **e**) and deactivation (**b**, **f**) kinetics. The measurements were conducted by consecutively applying increasing LED light intensities on a measured cell. (**c**, **d**, **g**, **h**) Summaries of half-life time constants (τ_H) of the fast (**c**, **g**) and slow (**d**, **h**) inactivation kinetics. Number over boxplots indicate the number of measured cells. Gray asterisks indicate statistical significance calculated using Friedman test (***P<0.001) (**a**, **b**, **e**, **f**) or Kruskal-Wallis test (*P<0.05, **P<0.01, ***P<0.001) (**c**, **d**, **g**, **h**). Black asterisks indicate significances calculated using Dunn's multiple comparison post hoc analysis (*P<0.05, **P<0.01, ***P<0.001) (**e**-**d**). **Related to Figures 1 and 2.**

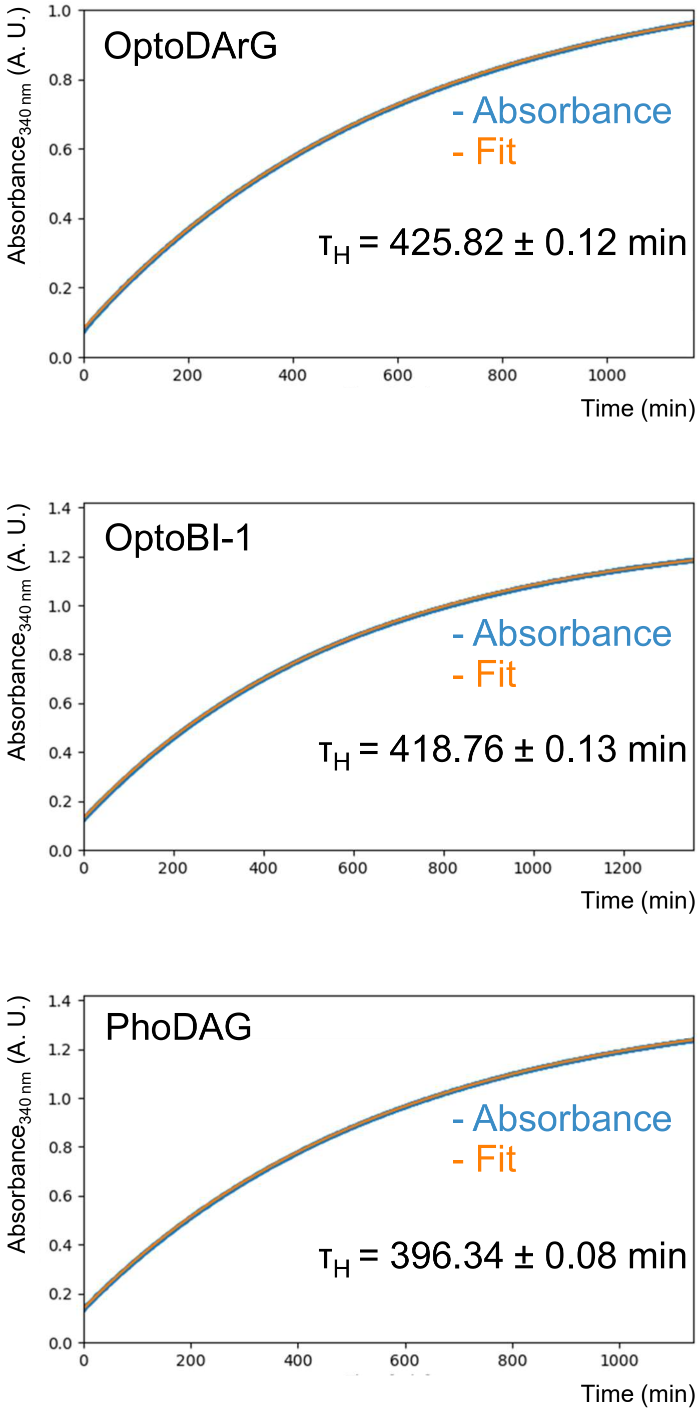

Supplemental Figure 5: Thermal relaxation of OptoDArG, OptoBI-1 and PhoDAG. OptoDArG. UV-Vis spectroscopy of OptoDArG, OptoBI-1 and PhoDAG (50 µM in 9:1 $(CD_3)_2SO$:D_2O). The compounds were preirradiated with 365 nm followed by relaxation in the dark at 37°C. The thermal relaxation was determined through UV-Vis analysis by measuring the time-dependent absorption at 340 nm with $A(t) = A_0 \cdot e^{\frac{-\ln(2)\cdot t}{\tau_H}} + c$. A_0: initial absorbance; c: maximal absorbance; τ_H: of half-life time constant. Fitted curve in orange and measured absorbance in blue. **Related to Figures 1-6.**

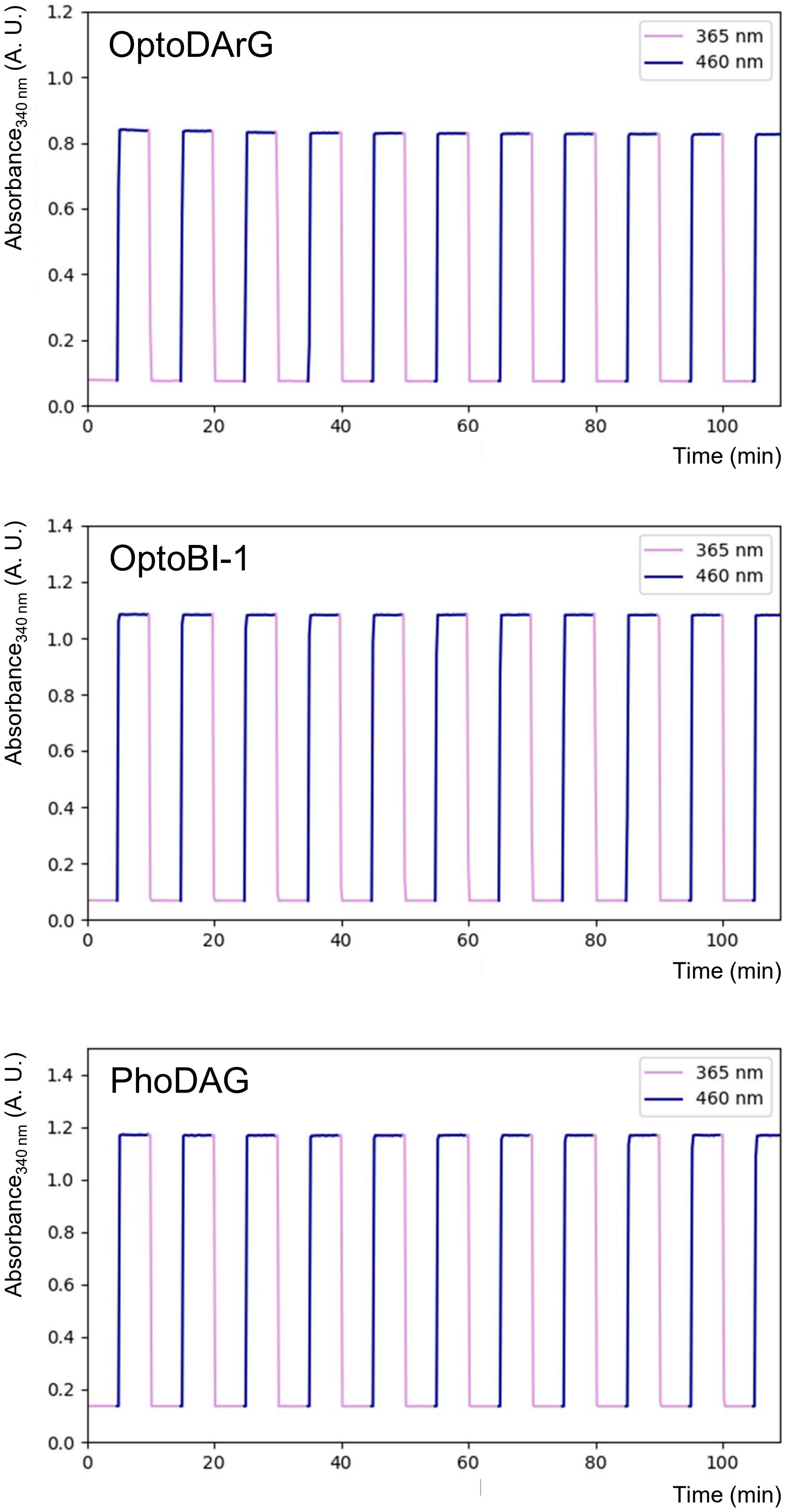

Supplemental Figure 6: Reversibility of photoswitching of OptoDArG, OptoBI-1 and PhoDAG. UV-Vis spectroscopic analysis of OptoDArG, OptoBI-1 and PhoDAG (50 µM in 9:1 $(CD_3)_2SO:D_2O$). Absorbance was measured at 340 nm at 22°C. Compounds were repeatedly illuminated with 365 nm and 460 nm light for 5 minutes each over a period of 110 minutes. **Related to Figures 1-6.**

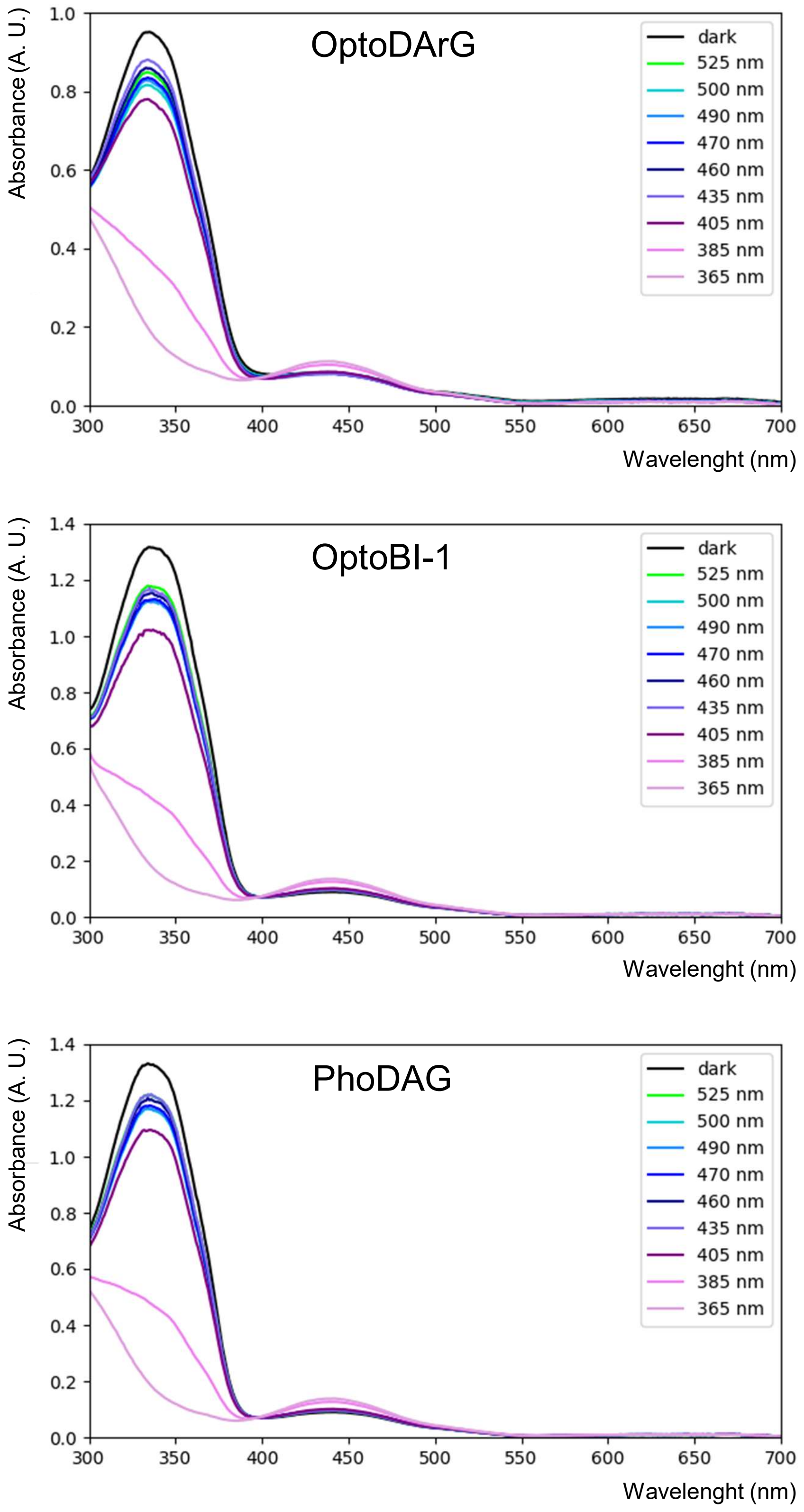

Supplemental Figure 7: PSS of OptoDArG, OptoBI-1 and PhoDAG with UV-Vis spectroscopy. UV-Vis spectroscopic analysis of OptoDArG, OptoBI-1 and PhoDAG (50 µM in 9:1 $(CD_3)_2SO:D_2O$). Spectral absorbance from 300 nm to 700 nm was measured at 22°C. The compounds were maximal thermally relaxed in the dark or illuminated with indicated wavelengths for 15 minutes. **Related to Figures 1-6.**

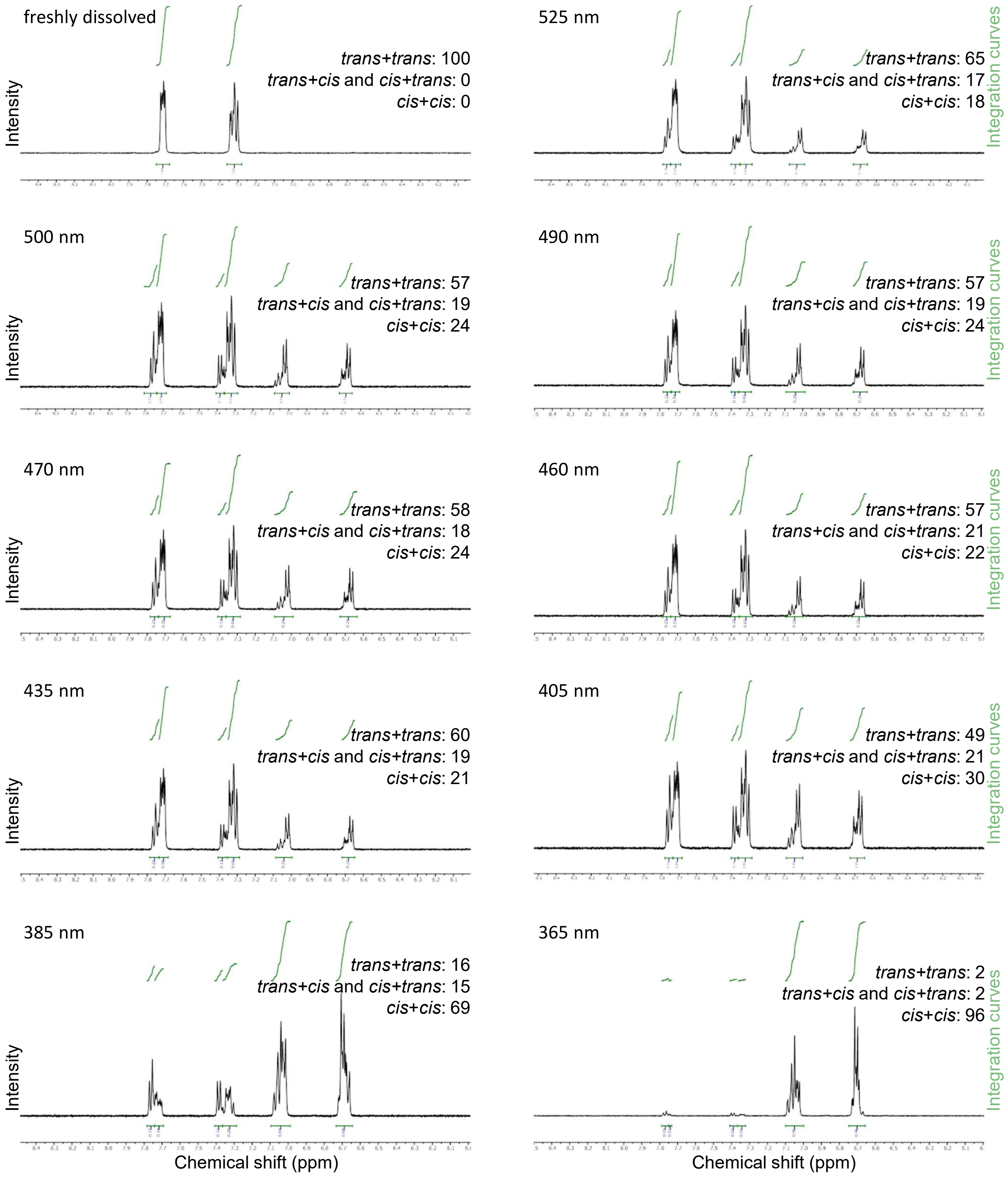

Supplemental Figure 8: ^{1}H-NMR spectroscopy with OptoDArG. ^{1}H-NMR spectroscopic analysis of OptoDArG (2.5 mM in 9:1 $(CD_3)_2SO:D_2O$) at 22°C. The compound was either freshly resolved or illuminated with indicated wavelengths for 15 minutes after maximal thermal relaxation. *Because OptoDArG has two azobenzene moieties, illumination with 435 nm and 365 nm light leads to a mixture of entirely trans (trans+trans), mixed (trans+cis* and *cis+trans), and entirely cis (cis+cis) conformational states.* **Related to Figures 1 and 3-6.**

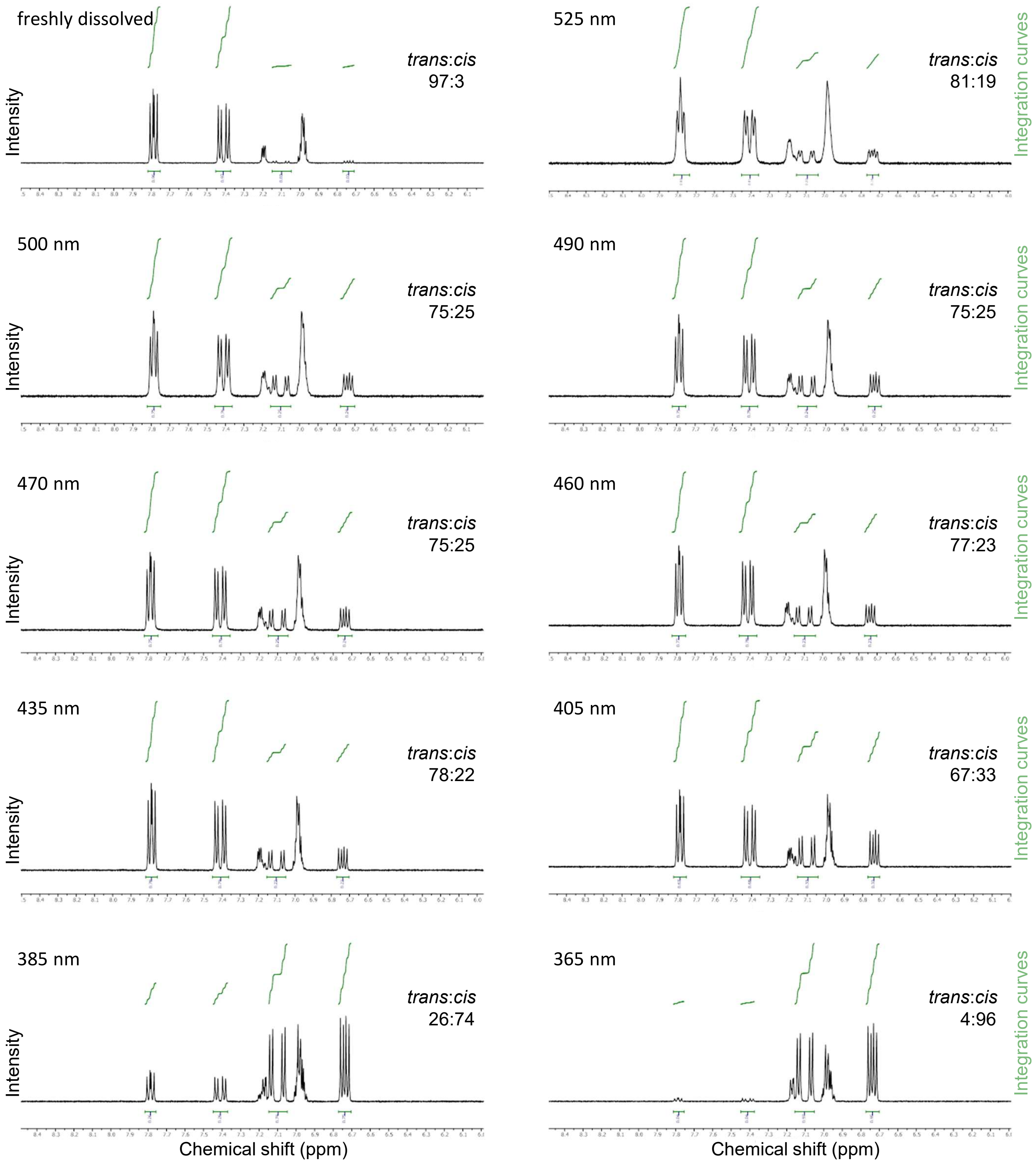

Supplemental Figure 9: ^{1}H-NMR spectroscopy with OptoBI-1. ^{1}H-NMR spectroscopic analysis of OptoBI-1 (2.5 mM in 9:1 $(CD_3)_2SO:D_2O$) at 22°C. The compound was either freshly resolved or illuminated with indicated wavelengths for 15 minutes after maximal thermal relaxation. **Related to Figures 2-6.**

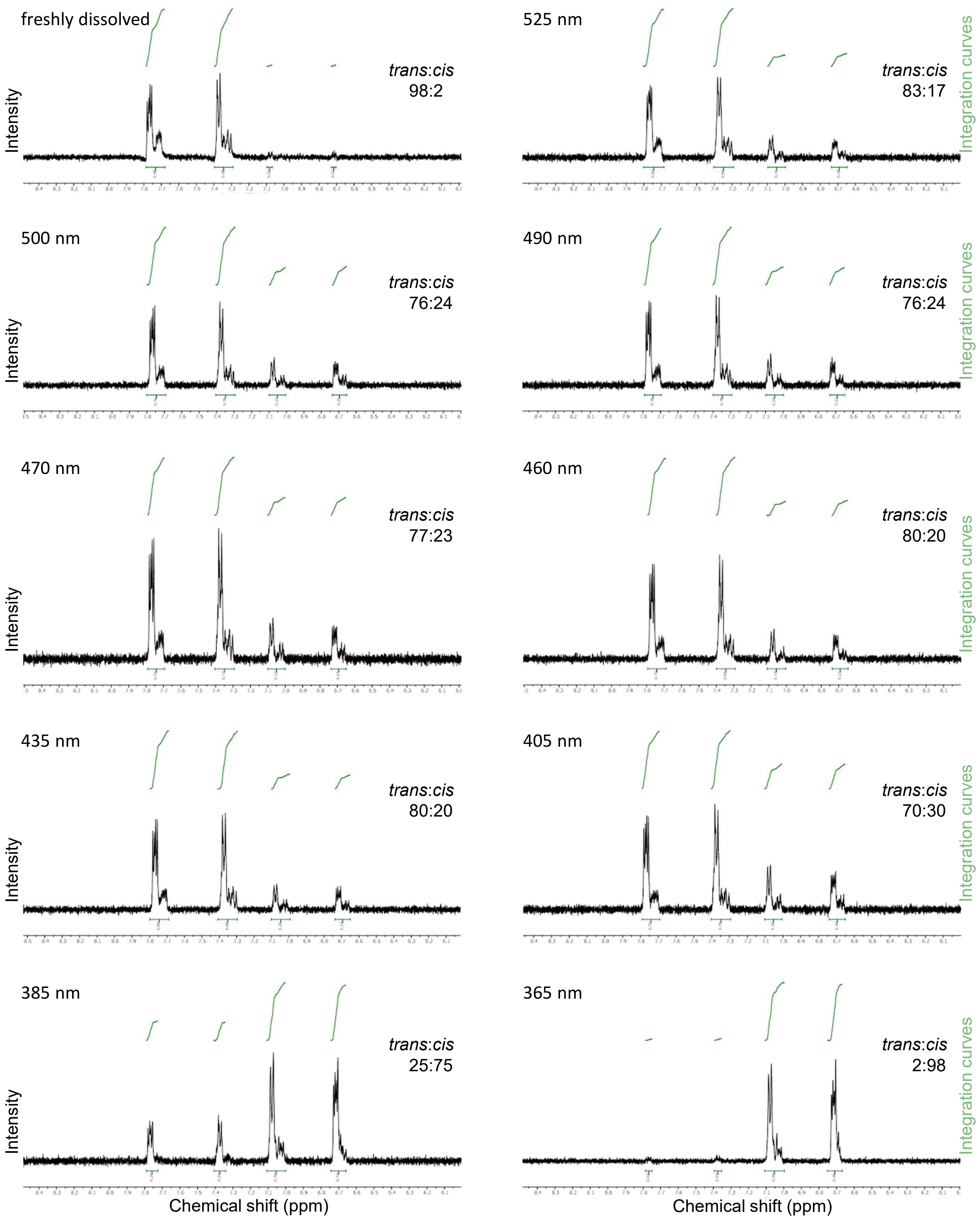

Supplemental Figure 10: ^{1}H-NMR spectroscopy with PhoDAG. ^{1}H-NMR spectroscopic analysis of PhoDAG (2.5 mM in 9:1 $(CD_3)_2SO{:}D_2O$) at 22°C. The compound was either freshly resolved or illuminated with indicated wavelengths for 15 minutes after maximal thermal relaxation. **Related to Figures 3-6.**

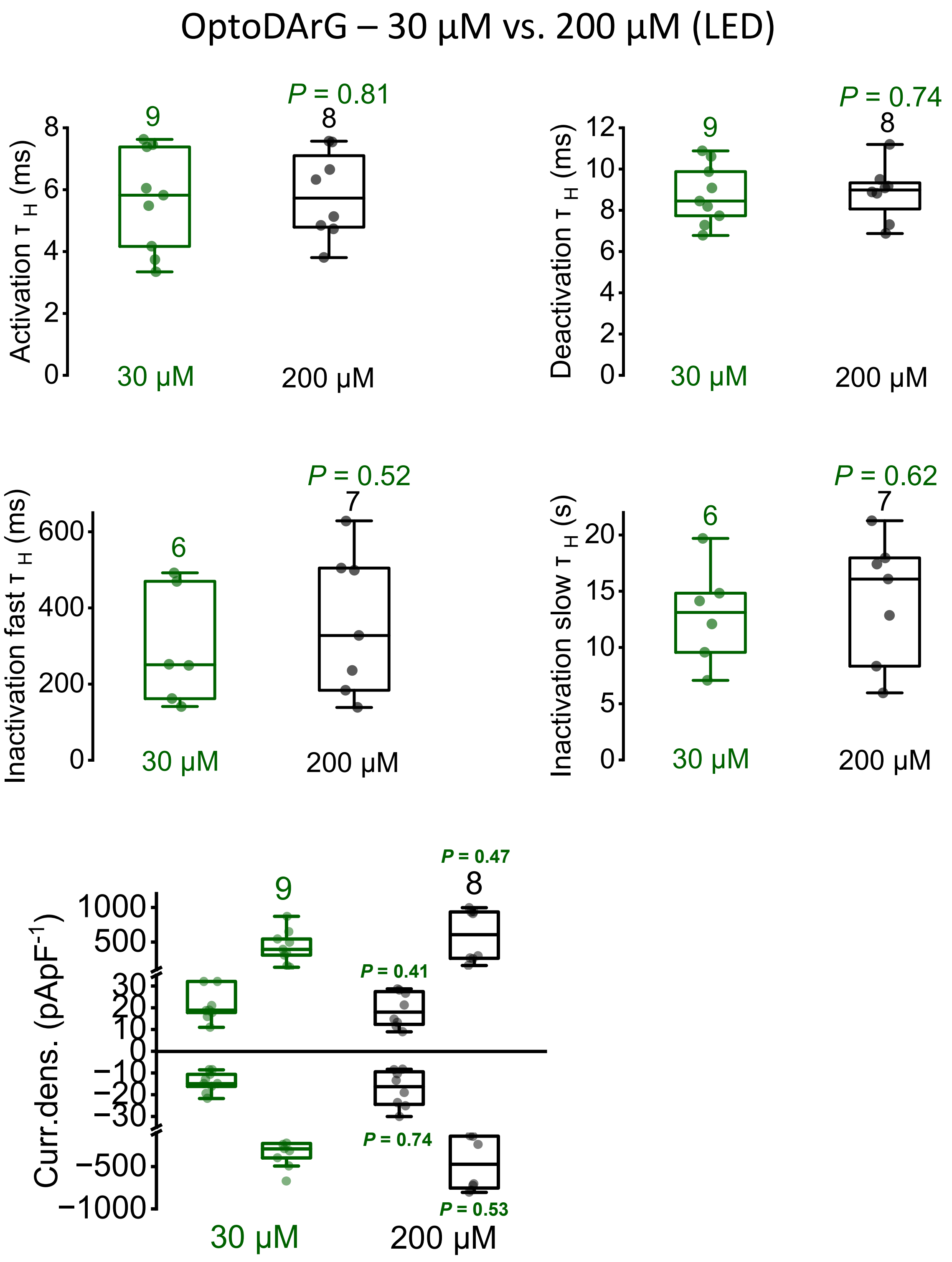

Supplemental Figure 11: Increased concentration of OptoDArG causes similar CD amplitudes and current kinetics. Whole-cell measurements of TRPC6 overexpressing HEK293T cells in the presence of 30 µM or 200 µM OptoDArG. Photoswitching was conducted with LED light with 100% light intensity. Summaries of half-life time constants (τ_H) of the activation, deactivation and of the fast and slow inactivation kinetics are displayed. Summary of current densities ('Curr. dens.') at ±100 mV in the presence of *trans*-OptoDArG (left boxplots at 30 and 200 µM OptoDArG) or of *cis*-OptoDArG (right boxplots at 30 and 200 µM OptoDArG) are displayed. Data are displayed as boxplots and interquartile ranges. Numbers over boxplots indicate number of measured cells.-Significant differences compared 30 µM OptoDArG were calculated using Mann Whitney *U* test. P values are displayed. **Related to Figures 1, 3 and 4.**

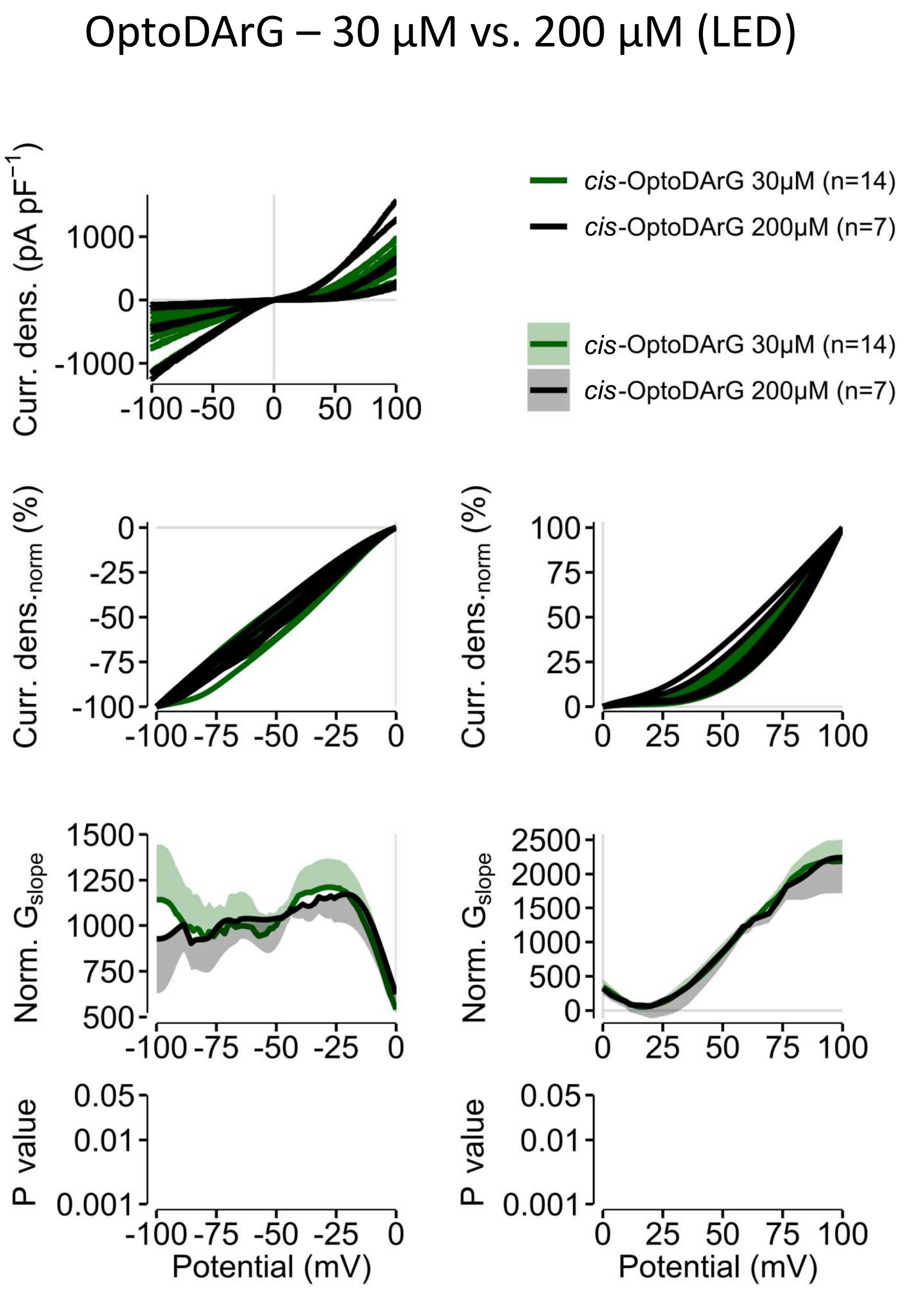

Supplemental Figure 12: Increased concentration of OptoDArG causes similar NSC curves. Whole-cell measurements of TRPC6 overexpressing HEK293T cells. Current density-voltage relations ('Curr. dens.') during application of blue light or UV light in the presence of 30 and 200 µM OptoDArG are displayed (above). The current density-voltage relations were smoothed and normalized ('Curr. $dens_{norm}$ (%)'). The calculated normalized slope conductance (NSC) ('Norm. G_{slope}') is displayed as mean ± SD. P values are calculated using Wilcoxon matched signed-rank test. **Related to Figures 5 and 6.**

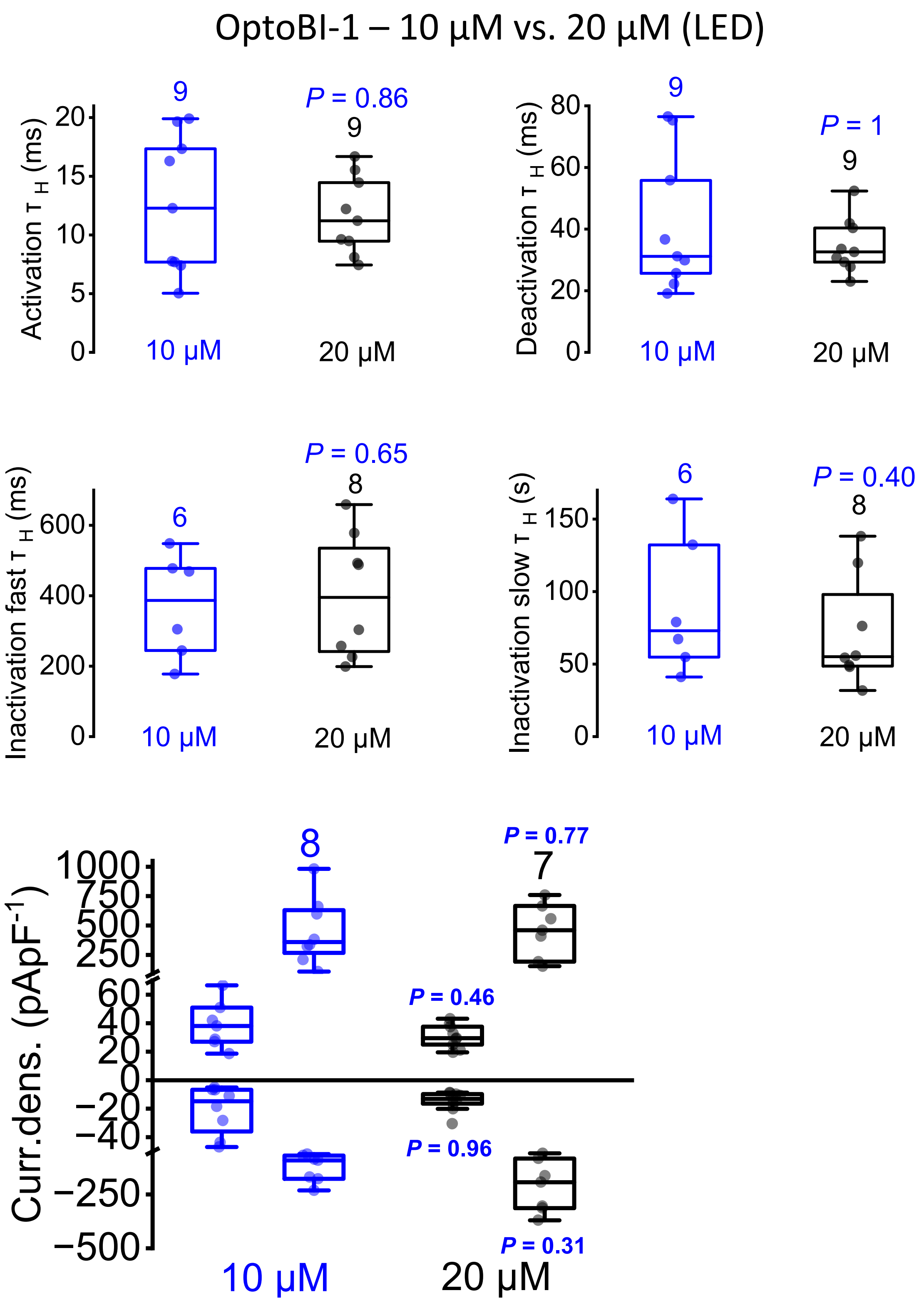

Supplemental Figure 13: Increased concentration of OptoBI-1 causes similar CD amplitudes and current kinetics. Whole-cell measurements of TRPC6 overexpressing HEK293T cells in the presence of 10 µM or 20 µM OptoBI-1. Photoswitching was conducted with LED light with 100% light intensity. Summaries of half-life time constants (τ_H) of the activation, deactivation and of the fast and slow inactivation kinetics are displayed. Summary of current densities (‘Curr. dens.’) at ±100 mV in the presence of *trans*-OptoBI-1 (left boxplots at 10 and 20 µM OptoBI-1) or of *cis*-OptoBI-1 (right boxplots at 10 and 20 µM OptoBI-1) are displayed. Data are displayed as boxplots and interquartile ranges. Numbers over boxplots indicate number of measured cells.-Significant differences compared 10 µM OptoBI-1 were calculated using Mann Whitney *U* test. P values are displayed. **Related to Figures 2-4.**

OptoBI-1 – 10 µM vs. 20 µM (LED)

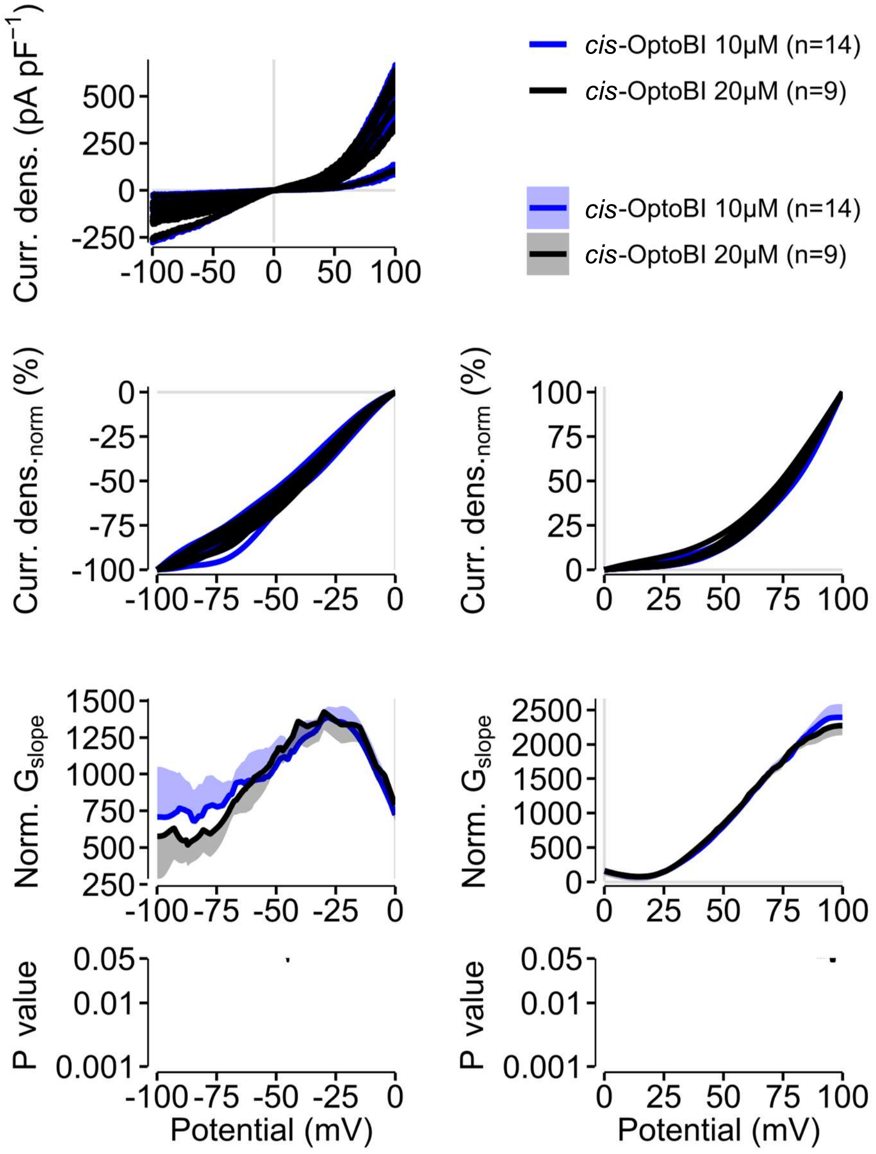

Supplemental Figure 14: Increased concentration of OptoBI-1 causes similar NSC curves. Whole-cell measurements of TRPC6 overexpressing HEK293T cells. Current density-voltage relations ('Curr. dens.') during application of blue light or UV light in the presence of 10 and 20 µM OptoBI-1 are displayed (above). The current density-voltage relations were smoothed and normalized ('Curr. $dens_{norm}$ (%)'). The calculated normalized slope conductance (NSC) ('Norm. G_{slope}') is displayed as mean ± SD. P values are calculated using Wilcoxon matched signed-rank test. **Related to Figures 5 and 6.**

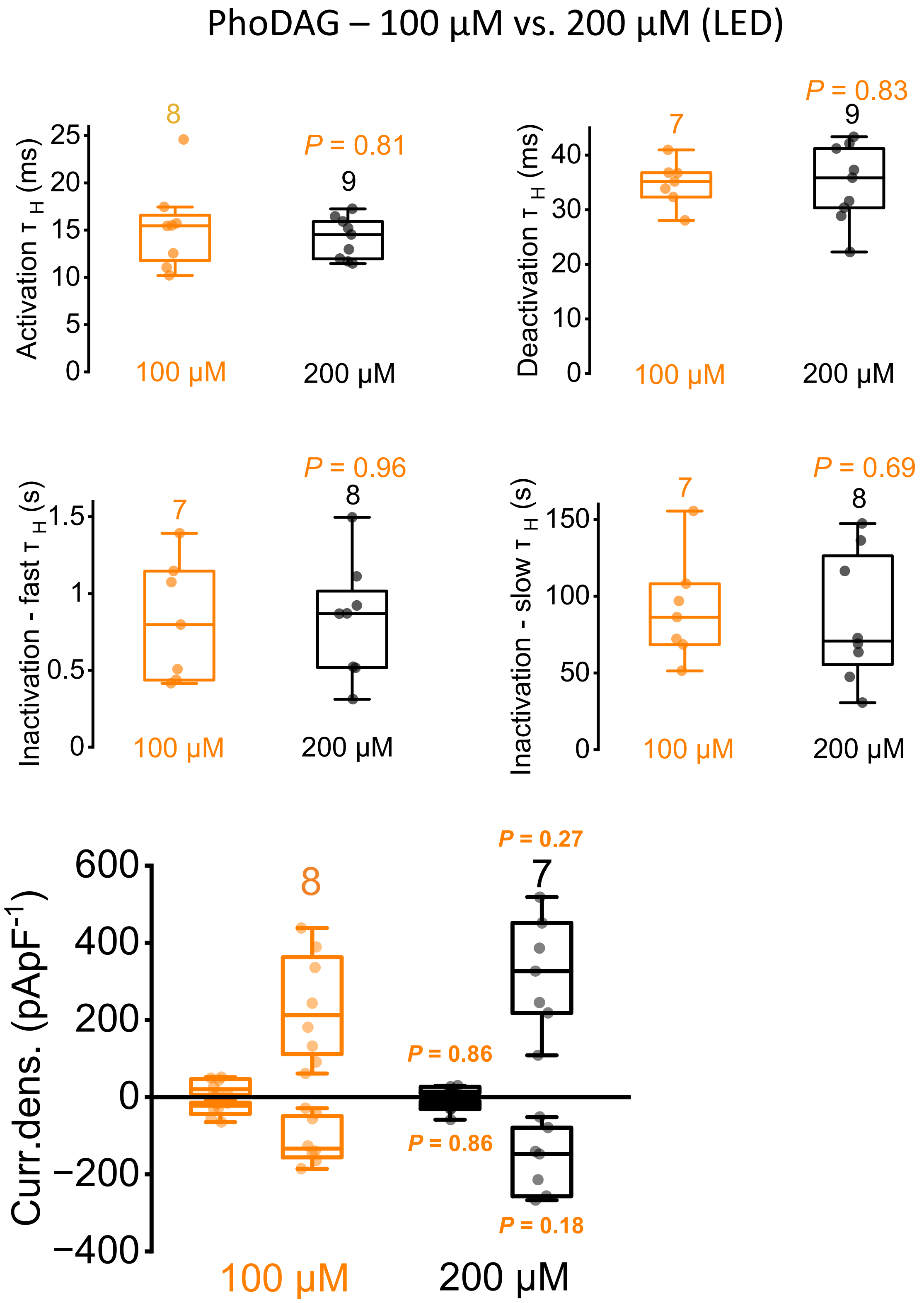

Supplemental Figure 15: Decreased concentration of PhoDAG causes similar CD amplitudes and current kinetics. Whole-cell measurements of TRPC6 overexpressing HEK293T cells in the presence of 100 µM or 200 µM PhoDAG. Photoswitching was conducted with LED light with 100% light intensity. Summaries of half-life time constants (τ_H) of the activation, deactivation and of the fast and slow inactivation kinetics are displayed. Summary of current densities ('Curr. dens.') at ±100 mV in the presence of *trans*-PhoDAG (left boxplots at 100 and 200 µM PhoDAG) or of *cis*-PhoDAG (right boxplots at 100 and 200 µM PhoDAG) are displayed. Data are displayed as boxplots and interquartile ranges. Numbers over boxplots indicate number of measured cells.–Significant differences compared to 200 µM PhoDAG were calculated using Mann Whitney *U* test. P values are displayed. **Related to Figures 3 and 4.**

PhoDAG – 100 µM vs. 200 µM (LED)

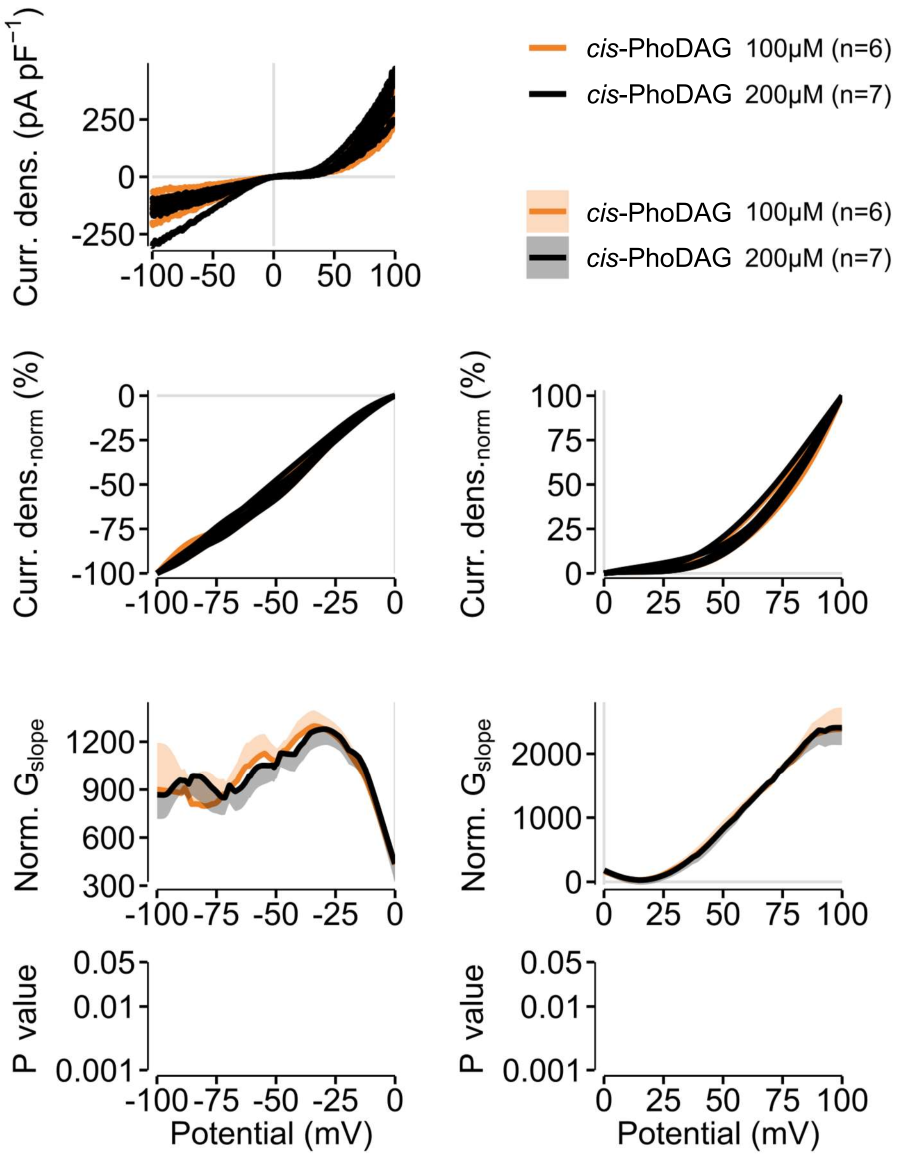

Supplemental Figure 16: Decreased concentration of PhoDAG causes similar NSC curves. Whole-cell measurements of TRPC6 overexpressing HEK293T cells. Current density-voltage relations ('Curr. dens.') during application of blue light or UV light in the presence of 100 and 200 µM PhoDAG are displayed (above). The current density-voltage relations were smoothed and normalized ('Curr. $dens_{norm}$ (%)'). The calculated normalized slope conductance (NSC) ('Norm. G_{slope}') is displayed as mean ± SD. P values are calculated using Wilcoxon matched signed-rank test. **Related to Figures 5 and 6.**

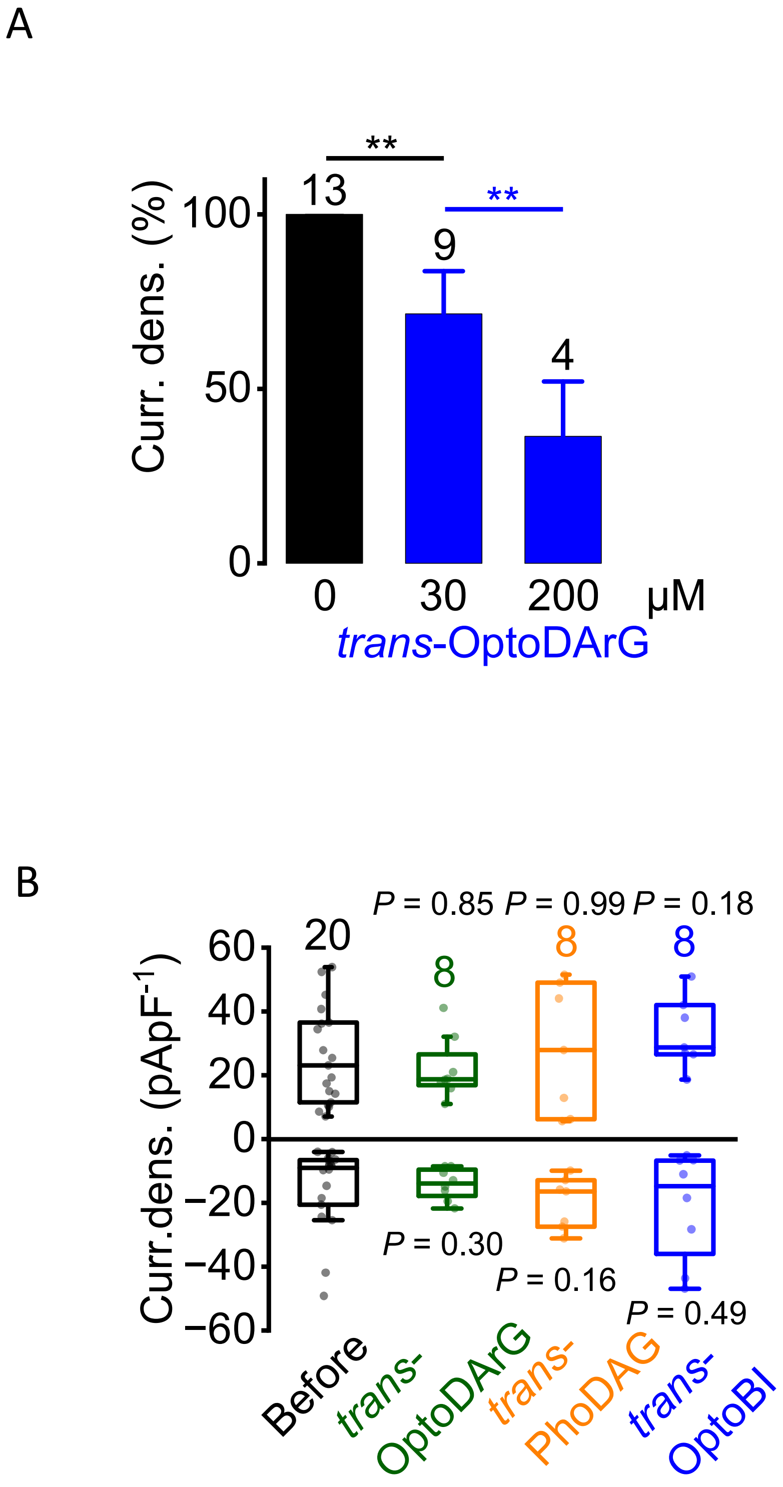

Supplemental Figure 17: Effect of *trans* isomers on TRPC3 or TRPC6 expressing HEK293T cells. (**a**) Summary of normalized current densities of basal TRPC3 outward currents at +100 mV in the absence (0 µM) or after wash in of 30 or 200 µM *trans*-OptoDArG. Data are displayed as median ± SD. Numbers over bars indicate number of measured cells. P values were calculated using Mann Whitney U test (**P<0.01; blue asterisks) or using one sample Wilcoxon test (**P<0.01; black asterisks). (**b**) Summary of current densities at ±100 mV of basal TRPC6 currents in the absence or in the presence of 30 µM *trans*-OptoDArG, 200 µM *trans*-PhoDAG or 10 µM *trans*-OptoBI. Data are displayed as boxplots and interquartile ranges. Numbers over boxplots indicate number of measured cells.-Significant differences compared to basal TRPC6 currents were calculated using Mann Whitney *U* test. P values are displayed. **Related to Figures 1-3.**

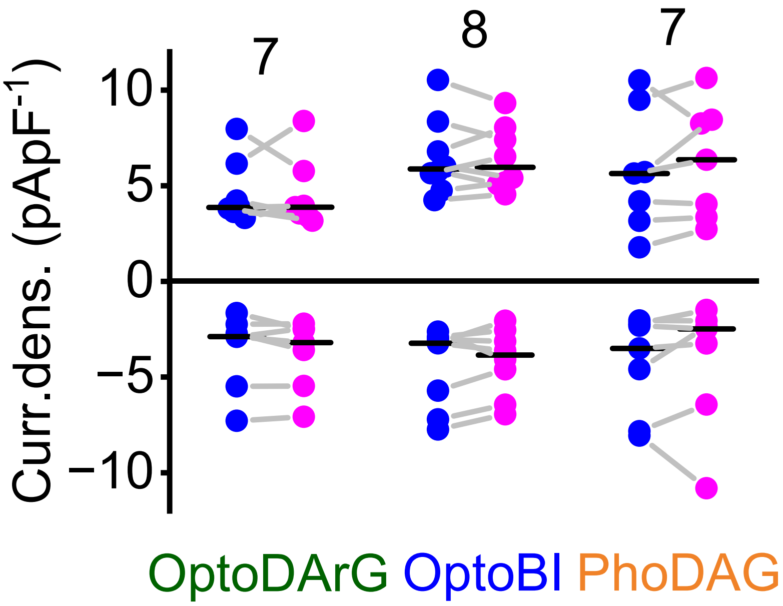

Supplemental Figure 18: Photoswitching of OptoDArG, OptoBI-1 and PhoDAG has no effect on non-transfected HEK293T cells. Summary of current densities of non-transfected HEK293T cells in the presence of OptoDArG, OptoBI-1 and PhoDAG. Blue dots indicate application of blue light and violet dots indicate application of UV light. Significant differences were calculated using Mann Whitney U test. Black horizontal lines represent medians. **Related to Figure 3.**

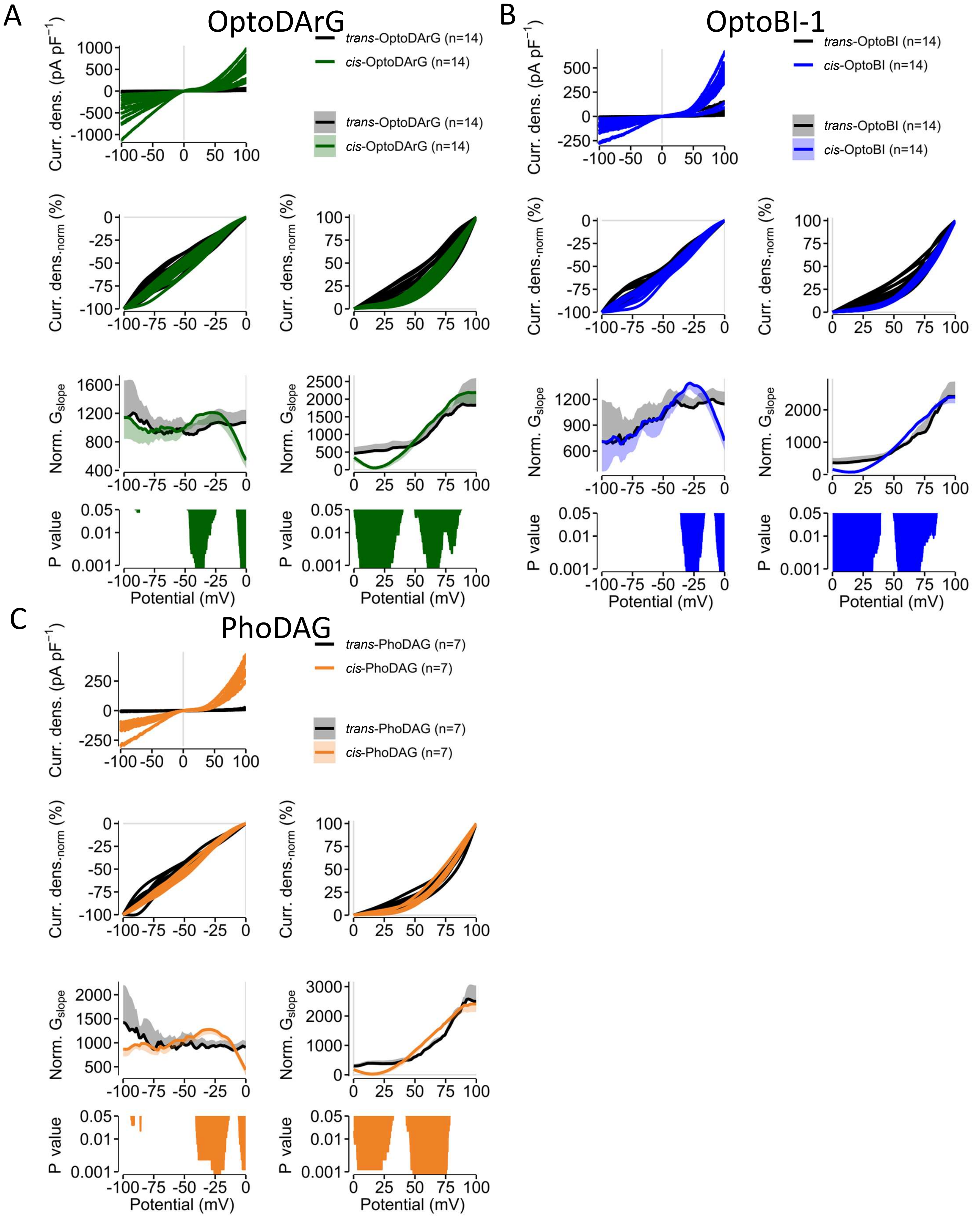

Supplemental Figure 19: Normalized slope conductance of TRPC6 currents induced by photopharmaceuticals. Whole-cell measurements of TRPC6 overexpressing HEK293T cells. (**a-c**) All current density-voltage relations ('Curr. dens.') during application of blue light or UV light in the presence of OptoDArG (**a**), OptoBI-1 (**b**) or PhoDAG (**c**) are displayed (above). The current density-voltage relations were smoothed and normalized ('Curr. $dens_{norm}$ (%)'). The calculated normalized slope conductance (NSC) ('Norm. G_{slope}') is displayed as mean ± SD. P values are calculated using Wilcoxon matched signed-rank test. **Related to Figure 6.**

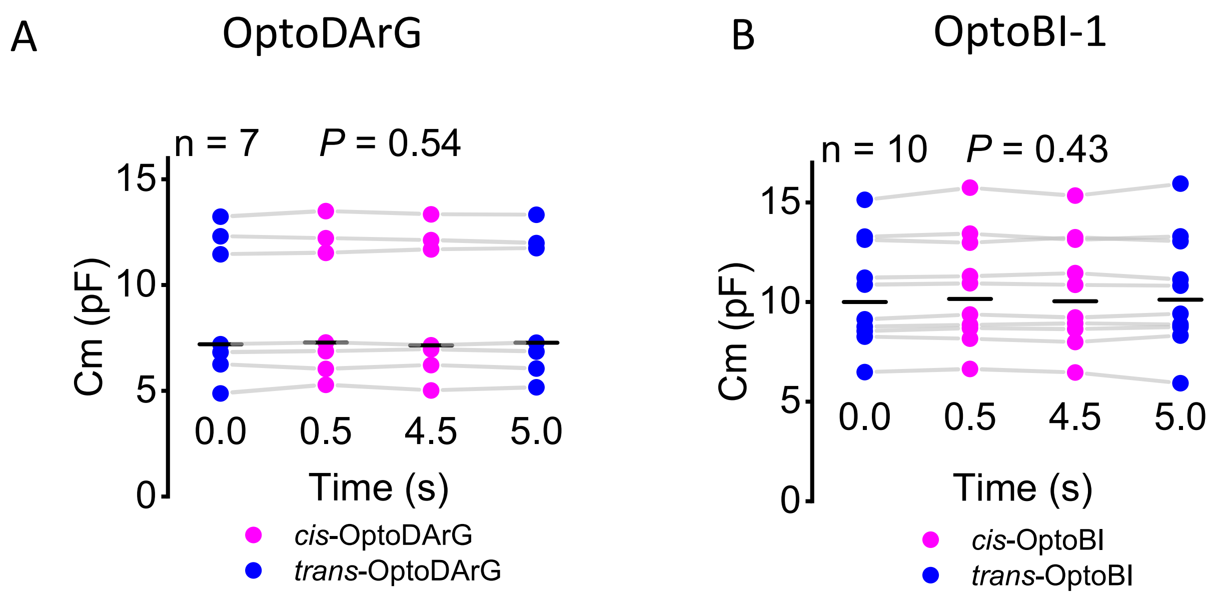

Supplemental Figure 20: Photoswitching of OptoDArG and OptoBI-1 has no effect on membrane capacitances. Changes of the membrane capacitance of TRPC6 expressing HEK293 cells in the presence of OptoDArG (**a**) or OptoBI-1 (**b**). The membrane capacitances (Cm) were determined in 500 ms steps during application of blue light before switching to UV light (0.0 s), after switching to UV light (0.5 and 4.5 s) and after switching to blue light (5.0 s). Significant differences were calculated using Friedman test. Gray horizontal lines represent medians. **Related to Figures 3.**
